# The impulsive behavior short scale–8 (I-8): A comprehensive validation of the English-language adaptation

**DOI:** 10.1371/journal.pone.0273801

**Published:** 2022-09-06

**Authors:** Katharina Groskurth, Désirée Nießen, Beatrice Rammstedt, Clemens M. Lechner

**Affiliations:** GESIS–Leibniz Institute for the Social Sciences, Mannheim, Germany; University of Queensland, AUSTRALIA

## Abstract

The Impulsive Behavior Short Scale–8 (I-8) measures the psychological construct of impulsivity with four subscales comprising two items each (completion time < 1 min). The aim of the present study was threefold: (1) to assess the psychometric properties (objectivity, reliability, and validity) of the English-language I-8; (2) to compare these psychometric properties with those of the original German-language source version of the scale; and (3) to test the cross-national comparability of the scale via measurement invariance tests. For this purpose, we used heterogeneous quota samples from the UK and Germany. Our results indicate that I-8 is a reliable and valid short scale with highly comparable psychometric properties across both language versions. In addition, I-8 showed a highly similar correlational pattern with various extraneous variables across the two nations. Furthermore, partial scalar invariance and full invariance of residual variances held, allowing the comparison of latent means and observed (co)variances across nations. I-8 lends itself as a measure of impulsive behavior especially in surveys in which assessment time is limited, such as in large-scale cross-national surveys.

## Introduction

Impulsivity is understood as the absence of behavioral control. Whiteside and Lynam proposed that four personality traits are related to impulsive behavior: urgency, lack of premeditation, lack of perseverance, and sensation seeking [1]. Impulsivity is associated with various negative behaviors (e.g., drug use) and with psychological and psychopathological disorders [2, 3]. Therefore, studies examining individual differences in impulsive behavior and its correlates need reliable and valid measures of the underlying traits. To meet this need, Kovaleva et al. developed and validated the German-language Skala Impulsives-Verhalten-8 (I-8) [4]. I-8 comprises four subscales: urgency, lack of premeditation, lack of perseverance, and sensation seeking. As each subscale consists of only two items, I-8 measures the four facets of impulsive behavior far more economically than other scales, for example, the 45-item UPPS scale developed by Whiteside and Lynam [1]. As an ultra-short scale, I-8 is particularly well-suited for surveys with time limitations or other questionnaire constraints. In the absence of a comparable measure in the English-language context, Kovaleva et al. adapted I-8 to English [4]. However, an empirical validation of this English-language adaptation—which we named Impulsive Behavior Short Scale–8—has hitherto been lacking. The present study aims to investigate the psychometric properties of the English-language adaptation and to test measurement invariance across the two language versions using quota samples from the UK and Germany.

## Theoretical background

Researchers typically conceive of impulsivity as a multidimensional construct. Numerous conceptualizations of impulsivity have been proposed, which are rooted in different personality theories [5–7] (for an overview, see [1]). However, the present section will focus only on the conceptualization of impulsivity proposed by Whiteside and Lynam [1], as the authors comprehensively integrated existing conceptualizations of the construct and based their model of impulsivity on the most prominent model of personality, the Big Five [8].

Following Whiteside and Lynam, four distinct personality facets that lead to impulsive behavior can be distinguished [1]: (1) *urgency*, that is “a tendency to commit rash or regrettable actions as a result of intense negative affect” (p. 677); (2) *lack of premeditation*, that is, acting without careful thinking and planning and without reflecting on the consequences of an act; (3) *lack of perseverance*, that is, the inability to remain focused on boring or difficult tasks or to ignore distracting stimuli; and (4) *sensation seeking*, that is, a tendency to engage in and enjoy exciting and risky activities.

These four facets are related to some of the Big Five personality traits [9]: Urgency encompasses neurotic aspects. Negative emotions, as part of the domain of Neuroticism, often result from impulsive actions that ignore long-term negative consequences. Individuals high in premeditation are thoughtful and rational; individuals high in perseverance have great self-discipline—dispositions that characterize the Big Five domain of Conscientiousness. Moreover, individuals who are high in sensation seeking engage in risky and exciting activities—a disposition that characterizes the domain of Extraversion [1, 10].

The four personality facets of impulsive behavior (i.e., urgency, lack of premeditation, lack of perseverance, and sensation seeking) can have several adverse consequences. For example, individuals high in urgency, lacking premeditation and perseverance, and seeking sensation are more likely to engage in negative and harmful behaviors such as problematic gambling [11]. Notably, certain personality facets specifically drive different kinds of (impulsive) behavior: A high lack of premeditation is strongly related to high delinquency [2, 12] and drug use [2]. High urgency is strongly associated with self-injury behavior [13], and high sensation seeking is associated with alcohol abuse (albeit only in individuals who are high in antisocial personality traits) [3, 14].

The four personality facets associated with impulsive behavior are also closely linked to several (sub)clinical and pathological cognitions and behaviors. For instance, because high urgency is an expression of the inability to regulate and cope with negative emotions, it is strongly associated with forms of psychopathology such as borderline personality disorder or pathological gambling [3]. Persons with attention deficit hyperactivity disorder (ADHD) typically have a high lack of premeditation [2].

To date, researchers have mainly investigated the associations between (sub)clinical and pathological cognitions/behaviors and urgency, lack of premeditation, lack of perseverance, and sensation seeking. By contrast, non-clinical associations have largely been ignored so far and thus must be investigated in more detail.

### Assessing impulsivity: The four-factor model

To assess the four personality facets related to impulsive behavior, Whiteside and Lynam developed the 45-item Urgency, Premeditation, Perseverance, and Sensation Seeking (UPPS) Impulsive Behavior Scale [1]. The UPPS scale includes items from various other impulsivity scales (e.g., Dickman’s Functional and Dysfunctional Impulsivity Scales [5]; see also [15]) and from personality inventories (e.g., Revised NEO Personality Inventory; NEO-PI-R; [16]). A series of exploratory factor analyses using data from the NEO-PI-R, eight widely used impulsivity measures, and additional “impulsiveness” items created by the investigators identified four personality facets that lead to impulsive behavior: urgency, lack of premeditation, lack of perseverance, and sensation seeking [1]. These four facets, which had previously been lumped together under the umbrella term “impulsivity,” were found to be theoretically distinct [1].

Several studies that tested the four-factor model of impulsivity provided evidence that the scale does indeed capture four distinct personal facets, and that these facets have different intercorrelations [3, 14, 17]. Whereas lack of premeditation and lack of perseverance were found to be substantially correlated (*r* = .45), all other intercorrelations were substantially lower (.00 *≤* |*r*| *≤* .29) [1, 18]. The rank order of the intercorrelations did not hold in every sample (which is unsurprising given the many low intercorrelations). For example, in a sample of 20-year-olds, Miller et al. replicated a strong correlation between lack of premeditation and lack of perseverance (*r* = .50), and they found similarly large correlations between these two facets and urgency (*r* = .56 and *r* = .41, respectively) [2]. Keye et al. replicated Miller et al.’s findings in a German student sample [10]. Correlations between lack of premeditation and lack of perseverance seemed to be generally strong across all of the aforementioned studies [1, 2, 10, 18].

Although the 45-item UPPS scale [1] is frequently used in research on impulsivity, it is too long for many surveys—especially for multi-thematic surveys (e.g., the German Socio-Economic Panel, SOEP) in which questionnaire space is very limited.

### Development of the I-8 scale

To enable researchers to survey impulsive behavior in contexts with limited time and resources, Kovaleva et al. developed the German-language Skala Impulsives-Verhalten-8 (I-8) [4]. The I-8 scale captures all four factors of the UPPS model with just two items each. The construction of I-8 was based mainly on two German-language versions of the UPPS scale developed by Keye et al. and Schmidt et al. [10, 17]. To construct the subscales urgency, lack of premeditation, and lack of perseverance, Kovaleva et al. used the results of Keye et al.’s and Schmidt et al.’s factor analyses [4, 10, 17]. They selected two items per subscale based on the level and stability of factor loadings across studies and theoretical aspects. To construct the subscale Sensation Seeking, they first created six items based on the UPPS items [1]. These six items were later reduced to two based on factor loading patterns and item total correlations in an initial construction sample [4]. The preliminary items of I-8 were cognitively pretested and subsequently modified based on the pretest results. Kovaleva et al. psychometrically validated the resulting I-8 in three large independent quota and random samples representing the adult population in Germany (aged 18 and older) [4]. The four-factor structure was corroborated, and the German-language I-8 was shown to be a psychometrically valid and reliable scale to measure traits that lead to impulsive behavior.

To enable I-8 to be used in English-language research, Kovaleva et al. adapted the scale to English [4]. When doing so, they followed the two-step procedure recommended by the International Test Commission [4, 19], which aligns with the TRAPD (Translation, Review, Adjudication, Pretesting, and Documentation) approach [20]: First, two professional translators (English native speakers) translated the items independently. One translation was done in British English, the other in American English. In the second phase of the adaptation process, a reconciliation meeting was held during which the proposed adaptations were discussed and revised in a group comprising experts on the psychological characteristics in question, the translators, and an expert on questionnaire adaptation.

However, the testing of the psychometric quality of the English-language adaptation of I-8 has remained a desideratum until now. The present study aims to fill this gap and, when doing so, to compare the English-language version of I-8 with the German source version and test the measurement invariance of the scale across the two languages. Measurement invariance is an important prerequisite for valid cross-cultural comparisons [21].

## Method

To establish the psychometric properties of the English-language adaptation of I-8, to compare them with those of the German-language source instrument, and to test the measurement invariance of the scale across the two languages, the respective versions were administered to respondents in a web-based survey conducted in parallel in the UK and Germany in January 2018 by the panel provider respondi AG using computer-assisted self-administered interviewing (CASI).

### Samples

For both nations, quota samples were drawn that represented the heterogeneity of the adult population in terms of age, sex, and educational attainment. The basis for the quotas for the UK and Germany was the latest 2011 German census (https://ergebnisse.zensus2011.de). The purpose of the research—to investigate the quality of several questionnaires—was explained to all respondents. Only native speakers of the respective languages were recruited. All respondents consented to participation in an anonymous online survey and were financially rewarded for their participation. A subsample was reassessed after approximately 3 to 4 weeks (median time intervals: 28 days in the UK and 20 days in Germany). According to the local legislation and requirements of the institution, our study on human participants did not require review and approval by an ethics committee as we collected data without any reference to the participants’ identity. The data collection was completely anonymous. We adhered to ethical standards comparable to the 1964 Declaration of Helsinki.

Only those respondents who did not abort the survey prematurely were included in our analysis. This resulted in a gross sample size of *N*_UK_ = 508 (retest: *N*_UK_ = 117) for the UK and *N*_DE_ = 513 (retest: *N*_DE_ = 125) for Germany. To ensure high data quality, we applied three criteria simultaneously. The first criterion was the ipsatized variance, that is, the within-person variance across items [22]. We excluded respondents with an ipsatized variance below 5%. The second criterion was the Mahalanobis distance of the respondent’s response vector from the average sample response vector [23]. Respondents within the upper 2.5% of the sample distribution of the Mahalanobis distance were excluded. The third criterion was the response time. Respondents who answered all items in less than 1 s per item, on average, were excluded. We chose relatively liberal heuristics for exclusion criteria to avoid erroneously excluding valid cases and thereby creating systematic bias in our data. These quality checks resulted in the exclusion of 8% of cases in both the UK and the German subsamples. The final net sample sizes were *N*_UK_ = 468 (retest: *N*_UK_ = 111) and *N*_DE_ = 474 (retest: *N*_DE_ = 117). Table 1 details the sample characteristics and their distribution. The target and real sample sizes per quota are listed in Table 2.

**Table 1:** Sample characteristics by nation.

	United Kingdom (UK)	Germany (DE)
*N*	468	474
Mean age in years (*SD*) [Range]	45.2 (14.5) [18–69]	44.0 (14.4) [18–69]
Proportion of women (%)	52.6	50.0
Educational attainment (%)		
Low	34.8	33.5
Intermediate	32.1	33.8
High	33.1	32.7

*Note*. The educational attainment levels were as follows: Low (UK) = Never went to school/Skills for Life/1–4 GCSEs A*–C or equivalent; Low (Germany) = No educational qualifications/lower secondary leaving certificate (*ohne Bildungsabschluss/Hauptschulabschluss*); Intermediate (UK) = Five or more GCSEs A*–C/vocational GCSE/GNVQ intermediate or equivalent; Intermediate (Germany) = Intermediate school leaving certificate (*mittlerer Schulabschluss*); High (UK) = Two or more A-levels or equivalent; High (Germany) = Higher education entrance qualification (*Fachhochschulreife/allgemeine Hochschulreife*).

**Table 2:** Quotas: Target and actual sample sizes for the UK and Germany.

Quota no.	Sex	Educational attainment	Age	Target *n* per quota	Actual *n* per quota	
					United Kingdom	Germany
1	Male	Low	18–29	22 (4.4%)	15 (3.2%)	15 (3.2%)
2			30–49	32 (6.5%)	27 (5.8%)	29 (6.1%)
3			50–69	41 (8.3%)	38 (8.1%)	40 (8.4%)
4		Intermediate	18–29	21 (4.3%)	17 (3.6%)	19 (4.0%)
5			30–49	32 (6.3%)	28 (6.0%)	30 (6.3%)
6			50–69	21 (4.1%)	21 (4.5%)	24 (5.1%)
7		High	18–29	18 (3.5%)	17 (3.6%)	17 (3.6%)
8			30–49	37 (7.4%)	33 (7.1%)	37 (7.8%)
9			50–69	26 (5.1%)	26 (5.6%)	26 (5.5%)
10	Female	Low	18–29	16 (3.2%)	15 (3.2%)	13 (2.7%)
11			30–49	24 (4.9%)	21 (4.5%)	22 (4.6%)
12			50–69	44 (8.8%)	47 (10.0%)	40 (8.4%)
13		Intermediate	18–29	22 (4.4%)	19 (4.1%)	21 (4.4%)
14			30–49	39 (7.8%)	36 (7.7%)	37 (7.8%)
15			50–69	29 (5.7%)	29 (6.2%)	29 (6.1%)
16		High	18–29	21 (4.1%)	21 (4.5%)	20 (4.2%)
17			30–49	36 (7.2%)	35 (7.5%)	36 (7.6%)
18			50–69	20 (3.9%)	23 (4.9%)	19 (4.0%)
			*N*	501 (100%)	468 (100%)	474 (100%)

*Note*. The educational attainment levels were as follows: Low (UK) = Never went to school/Skills for Life/1–4 GCSEs A*–C or equivalent; Low (Germany) = No educational qualifications/lower secondary leaving certificate (*ohne Bildungsabschluss/Hauptschulabschluss*); Intermediate (UK) = Five or more GCSEs A*–C/vocational GCSE/GNVQ intermediate or equivalent; Intermediate Germany) = Intermediate school leaving certificate (*mittlerer Schulabschluss*); High (UK) = Two or more A-levels or equivalent; High (Germany) = Higher education entrance qualification (*Fachhochschulreife/allgemeine Hochschulreife*). The target *n* per quota was calculated based on the latest German census (2011; https://ergebnisse.zensus2011.de).

The real *n* per quota was calculated after data cleansing.

### Materials

I-8 consists of eight items measuring four personality facets that lead to impulsive behavior: urgency, lack of premeditation, lack of perseverance, and sensation seeking. The English-language I-8 items are displayed in Table 3 and in the S1 Appendix. The original German-language I-8 items [4] are provided in the S2 Appendix. Items 1 and 2 belong to the urgency subscale, Items 3 and 4 to the lack of premeditation subscale, Items 5 and 6 to the lack of perseverance subscale, and Items 7 and 8 to the sensation seeking subscale. All items are answered using a 5-point rating scale ranging from 1 (*does not apply at all*) to 5 (*applies completely*). Whereas Items 1, 2, 7, and 8 are positively worded, Items 3, 4, 5 and 6 must be recoded so that they reflect the constructs lack of premeditation and lack of perseverance.

**Table 3:** Items of I-8.

No.	Subscale	Item
1	Urgency	Sometimes I do things impulsively that I should not do.
2		I sometimes do things to cheer myself up that I later regret.
3	Lack ofPremeditation	I usually think carefully before I act. (R)
4		I usually consider things carefully and logically before I make up my mind. (R)
5	Lack of Perseverance	I always bring to an end what I have started. (R)
6		I plan my schedule so that I get everything done on time. (R)
7	Sensation Seeking	I am willing to take risks.
8		I am happy to take chances.

*Note*. The general instructions are as follows: “The following statements may apply more or less to you. To what extent do you think each statement applies to you personally?” (R) indicates reverse coded items.

We validated I-8 by evaluating its nomological network, which consists of a broad range of non-clinical correlates. Whereas the associations between the I-8 factors and (a) the Big Five domains of personality were clearly established within the framework of the development of the German-language scale [14], associations with other individual-difference constructs were less clear and had not been established to date. Based on the definition of the four factors of the I-8 scale, we tentatively assumed the following: (b) Self-esteem is independent of the four factors; (c) higher self-efficacy is associated with a higher level of Premeditation, Perseverance, and Sensation Seeking; (d) higher (internal) locus of control is related to lower urgency, lack of premeditation, lack of perseverance, and sensation seeking; (e) higher life satisfaction is associated with lower urgency and with a higher level of premeditation [24]; (f) high risk proneness is related to high sensation seeking. We assumed correlations with (g) general health to be minor, acknowledging that higher urgency, lack of premeditation, lack of perseverance, and sensation seeking are related to many psychological disorders but not to physiological disorders. We further assumed that (h) socially desirable responding as a form of response bias is present when answering the I-8 items. Individuals commonly perceive impulsive behavior—and thus also personality facets that lead to such behavior—to be socially undesirable (as evidenced indirectly by the fact that the facets are associated with harmful behaviors). Accordingly, the respective language versions of the following short scales were also administered as part of the survey:

- The extra-short form of the Big Five Inventory–2 (BFI-2-XS; English version: [25]; German version: [26])
- The Rosenberg Self-Esteem Scale (RSES; English version: [27]; German version: [28])
- The General Self-Efficacy Short Scale–3 (GSE-3) [29]/Allgemeine Selbstwirksamkeit Kurzskala (ASKU) [30]
- The Internal–External Locus of Control Short Scale–4 (IE-4; [31])/Internale–Externale-Kontrollüberzeugung–4 [32]
- The General Life Satisfaction Short Scale (L-1) [33]/Kurzskala zur Erfassung der Allgemeinen Lebenszufriedenheit [34]
- The Risk Proneness Short Scale (R-1) [35]/Kurzskala zur Erfassung der Risikobereitschaft [36]
- The single-item European Social Survey question measuring self-reported general health [37]
- The Social Desirability–Gamma Short Scale (KSE-G) [38]/Kurzskala Soziale Erwünschtheit–Gamma [39]

And finally, we also investigated the associations between respondents’ sociodemographic characteristics and different values on the four factors of I-8. Thus, the survey included several sociodemographic variables: employment status, income, educational attainment, age, and sex. Employment status was surveyed with the following nominal categories: (1) employed, (2) self-employed, (3) out of work and looking for work, (4) out of work but not currently looking for work, (5) doing housework, (6) pupil/student, (7) apprentice/intern, (8) retired, and (9) none of the above. We recoded the variable into (1) unemployed (i.e., out of work and looking for work or out of work but not currently looking for work), and (2) employed (i.e., employed or self-employed). All remaining categories were regarded as missing values.

### Analytical strategy

The present study aimed to validate the English-language I-8 in the UK and to compare its psychometric properties with those of the German-language source version administered in parallel in Germany. We analyzed the descriptive statistics and psychometric quality criteria—objectivity, reliability, and validity—in both language versions. Additionally, we examined the cross-national comparability of the scale via measurement invariance tests.

We conducted all statistical analyses with R (version 3.6.3), using mainly the R packages lavaan [40], psych [41], and semTools [42]. We have made the analysis code available in the S3 Appendix.

## Results

### Descriptive statistics and reference ranges

Table 4 shows descriptive statistics—the mean, standard deviation, skewness, and excess kurtosis of each of the eight items of I-8—for the English-language adaptation and the German-language source version. We found no substantial divergence of descriptive statistics across nations. Table 5 provides detailed information on reference ranges and accompanying descriptive statistics of I-8 for the total population and clustered by sex and age.

**Table 4:** Descriptive statistics of the I-8 items by nation.

	*M*		*SD*		Skewness		Excess Kurtosis	
(No.) Item	UK	DE	UK	DE	UK	DE	UK	DE
Urgency								
(1) Sometimes I do things impulsively that I should not do.	2.62	2.83	1.16	0.99	0.45	0.23	−0.49	−0.21
(2) I sometimes do things to cheer myself up that I later regret.	2.53	2.44	1.23	1.06	0.41	0.59	−0.81	−0.20
Lack of Premeditation								
(3) I usually think carefully before I act. (R)	2.49	2.26	1.03	0.82	0.39	0.50	−0.54	0.08
(4) I usually consider things carefully and logically before I make up my mind. (R)	2.40	2.33	1.00	0.86	0.56	0.38	−0.17	−0.17
Lack of Perseverance								
(5) I always bring to an end what I have started. (R)	2.42	1.89	1.02	0.81	0.56	0.81	−0.13	0.63
(6) I plan my schedule so that I get everything done on time. (R)	2.48	2.16	1.07	0.94	0.50	0.75	−0.37	0.42
Sensation Seeking								
(7) I am willing to take risks.	2.84	2.97	1.19	1.04	−0.01	0.10	−0.92	−0.54
(8) I am happy to take chances.	2.91	3.08	1.15	1.02	0.03	0.07	−0.79	−0.68

*Note*. Response options ranged from 1 (*does not apply at all*) to 5 (*applies completely*). UK = United Kingdom (*N =* 468); DE = Germany (*N =* 474). (R) denotes items that were recoded.

**Table 5:** Reference ranges of the I-8 scale scores for the total population and separately for sex and age cohorts.

	*M*		*SD*		Skewness		Excess Kurtosis	
	UK	DE	UK	DE	UK	DE	UK	DE
Urgency								
Total population	2.58	2.63	1.10	0.94	0.55	0.47	−0.48	−0.06
Male [*n*_UK_ = 222; *n*_DE_ = 237]	2.67	2.60	1.07	0.90	0.42	0.42	−0.52	−0.04
Female [*n*_UK_ = 246; *n*_DE_ = 237]	2.49	2.67	1.12	0.98	0.67	0.48	−0.39	−0.15
18−29 [*n*_UK_ = 104; *n*_DE_ = 105]	2.94	2.87	1.17	1.00	0.10	0.31	−0.89	−0.47
30−49 [*n*_UK_ = 180; *n*_DE_ = 191]	2.65	2.70	1.10	0.94	0.48	0.46	−0.59	−0.25
50−69 [*n*_UK_ = 184; *n*_DE_ = 178]	2.30	2.42	0.98	0.85	0.87	0.46	0.37	0.36
Lack of Premeditation								
Total population	2.44	2.29	0.94	0.78	0.40	0.46	−0.31	0.03
Male [*n*_UK_ = 222; *n*_DE_ = 237]	2.39	2.22	0.88	0.74	0.45	0.46	−0.03	−0.04
Female [*n*_UK_ = 246; *n*_DE_ = 237]	2.49	2.37	1.00	0.82	0.33	0.42	−0.54	−0.02
18−29 [*n*_UK_ = 104; *n*_DE_ = 105]	2.52	2.36	0.95	0.86	0.22	0.13	−0.67	−0.90
30−49 [*n*_UK_ = 180; *n*_DE_ = 191]	2.36	2.32	0.92	0.79	0.52	0.66	0.05	0.55
50−69 [*n*_UK_ = 184; *n*_DE_ = 178]	2.48	2.22	0.96	0.72	0.37	0.41	−0.42	−0.02
Lack of Perseverance								
Total population	2.45	2.03	0.91	0.75	0.45	0.73	−0.09	0.67
Male [*n*_UK_ = 222; *n*_DE_ = 237]	2.48	2.04	0.87	0.72	0.45	0.65	0.05	0.33
Female [*n*_UK_ = 246; *n*_DE_ = 237]	2.42	2.02	0.94	0.79	0.46	0.79	−0.23	0.84
18−29 [*n*_UK_ = 104; *n*_DE_ = 105]	2.34	2.17	0.91	0.88	0.52	0.54	−0.20	−0.41
30−49 [*n*_UK_ = 180; *n*_DE_ = 191]	2.40	2.04	0.90	0.80	0.58	0.88	0.49	0.94
50−69 [*n*_UK_ = 184; *n*_DE_ = 178]	2.56	1.93	0.91	0.60	0.29	0.10	−0.52	−0.57
Sensation Seeking								
Total population	2.87	3.03	1.14	0.99	0.02	0.11	−0.84	−0.58
Male [*n*_UK_ = 222; *n*_DE_ = 237]	3.08	3.12	1.08	0.96	−0.08	−0.07	−0.64	−0.50
Female [*n*_UK_ = 246; *n*_DE_ = 237]	2.68	2.93	1.15	1.01	0.15	0.29	−0.96	−0.56
18−29 [*n*_UK_ = 104; *n*_DE_ = 105]	3.41	3.46	1.03	0.92	−0.32	−0.24	−0.59	−0.48
30−49 [*n*_UK_ = 180; *n*_DE_ = 191]	2.95	3.02	1.08	1.01	−0.14	0.10	−0.67	−0.60
50−69 [*n*_UK_ = 184; *n*_DE_ = 178]	2.49	2.78	1.12	0.93	0.43	0.33	−0.61	−0.24

*Note*. Answer options ranged from 1 (*does not apply at all*) to 5 (*applies completely*). UK = United Kingdom (*N =* 468); DE = Germany (*N =* 474).

### Objectivity

I-8 can be applied, evaluated, and interpreted objectively. It contains fixed instructions, a fixed item order, and a fixed number of labeled response options (objectivity of application). Further, I-8 is accompanied by strict rules on modeling and sum score derivation (objectivity of evaluation). Additionally, reference values (i.e., descriptive statistics) have been provided (objectivity of interpretation).

### Reliability

We calculated McDonald’s omega [43, 44] and test–retest stability to investigate the reliability of the four subscales of I-8, namely, urgency, lack of premeditation, lack of perseverance, and sensation seeking. Table 6 shows the reliability estimates. Following the classification proposed by Kline [45], who postulated that reliability coefficients are excellent around .90, very good around .80, and adequate around .70, all subscales of I-8 showed adequate to very good internal consistency in both the UK and Germany. I-8 was relatively stable across a 3- to 4-week interval in both nations. Test–retest stability was lowest for the urgency subscale in the UK and the lack of premeditation subscale in Germany.

**Table 6:** Reliability estimates of I-8.

	ω		*r*_tt_ [95% CI]	
	UK	DE	UK	DE
Urgency	.82	.80	.54 [.39, .66]	.69 [.59, .78]
Lack of Premeditation	.84	.84	.67 [.56, .76]	.45 [.30, .59]
Lack of Perseverance	.67	.65	.68 [.57, .77]	.61 [.48, .71]
Sensation Seeking	.95	.91	.72 [.61, .80]	.77 [.69, .84]

*Note*. UK = United Kingdom (*N =* 468; retest: *N* = 111); DE = Germany (*N =* 474; retest: *N* = 117); CI = confidence interval.

Especially considering the small number of items (i.e., two) per subscale, internal consistencies were sufficiently high for research purposes [46, 47]. The test–retest stabilities showed medium to large stabilities across a 1-month interval, and can also be deemed sufficient, especially because they were influenced not only by unreliability but also by true state fluctuations in impulsive behavior. The internal consistencies were similar to those found in other studies (e.g., Cronbach’s alpha of a short French version of the UPSS-P scale ranged from .70 to .84 [48], and Cronbach’s alpha of a short Japanese version of that scale ranged from .65 to .79 [49]—Cronbach’s alpha is practically identical to omega from Table 6 as the I-8 model is an essentially tau-equivalent one as shown in the Validity section, see also [50]). By contrast, the test–retest stabilities were lower compared to other studies (e.g., the test–retest stabilities of the aforementioned short French version of the UPSS-P scale after 2 weeks ranged from .84 to .92 [48], and the test–retest stabilities of the short Japanese version of that scale after 2 weeks ranged from .74 to .80 [49]).

### Validity

We assessed the factorial validity and the nomological network of I-8 to evaluate its validity in the UK and Germany.

#### Factorial validity

I-8 conceptualizes four distinctive personality facets related to impulsive behavior—namely, urgency, lack of premeditation, lack of perseverance, and sensation seeking. I-8 should reflect these four facets with two items each. We tested this assumption by fitting a four-dimensional confirmatory factor analysis (CFA) model. The factors were allowed to covary. We assessed model fit via heuristics for fit indices. In line with common guidelines, we judged model fit as adequate if the confirmatory fit index (CFI) was .950 or higher, the root mean square error of approximation (RMSEA) was .060 or lower, and the standardized root mean residual (SRMR) was .080 or lower [51]. We fixed the first loading of each factor to one and the first intercept to zero to identify the model. We estimated our model with robust maximum likelihood estimation (MLR).

**Fig 1:**
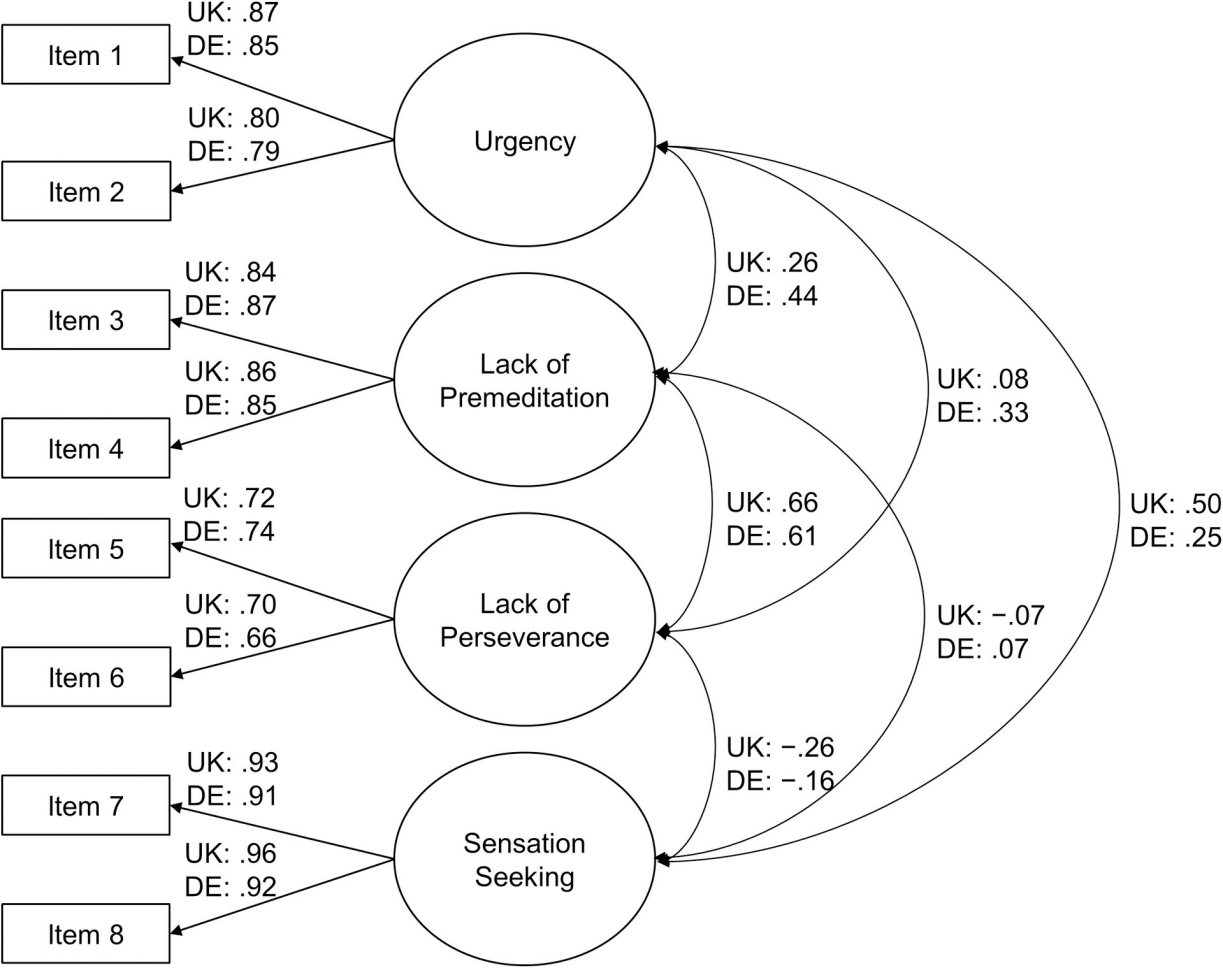
Four-factor essentially tau-equivalent measurement model of I-8 with standardized coefficients. *Note*. We omitted residual terms for clarity. *N*_UK_ = 468; *N*_DE_ = 474.

The proposed four-dimensional model fit well in both nations—UK/Germany: χ^2^(14) = 13.29/23.49, *p* = .504/053, CFI = 1.000/.992, RMSEA = .000/.038, SRMR = .016/.022, Bayesian information criterion (BIC) = 9,695/8,881. Brosseau-Liard et al. and Brosseau-Liard and Savalei found that applied robust corrections of the fit indices using MLR were not theoretically justified [52, 53]. Thus, they supported new corrections, which were implemented as so-called robust CFI and robust RMSEA values in R/lavaan (UK/Germany: robust CFI = 1.000/.993, robust RMSEA = .000/.039). As the proposed four-dimensional model fit exceptionally well, we tested a more restrictive model in which we imposed equality constraints on the factor loadings of the two items of each subscale/latent variable. The proposed four-dimensional model, which has freely estimated factor loadings, is called “tau-congeneric.” In contrast, the more restrictive model is called “essentially tau-equivalent” because it has identical factor loadings for all indicators [54]. The essentially tau-equivalent model fit as well as the congeneric one; thus, we accepted the former instead of the latter—UK/Germany: χ^2^(18) = 15.11/25.77, p = .654/.105, CFI = 1.000/.993, robust CFI = 1.000/.994, RMSEA = .000/.030, robust RMSEA = .000/.032, SRMR = .016/.026, BIC = 9,672/8,859. Fig 1 shows the final essentially tau-equivalent measurement model.

In the essentially tau-equivalent model shown in Fig 1, the correlations of the subscales were as follows: We observed the strongest correlation between lack of premeditation and lack of perseverance (UK/DE: *r* = .66/.61), which is in line with earlier research [2, 10, 18]. Other correlations between subscales were lower. Urgency correlated positively with lack of premeditation (UK/DE: *r* = .26/.44) and lack of perseverance (UK/DE: *r* = .08/.33). Urgency also correlated positively with sensation seeking (UK/DE: *r* = .50/.25). The correlation between lack of premeditation and sensation seeking was comparatively small (UK/DE: *r* = −.07/.07). Lack of perseverance correlated negatively with sensation seeking (UK/DE: *r* = −.26/−.16). Because theoretically [1] and empirically there is no general construct of impulsivity, as evidenced by the substantially varying correlations across subscales, we do not recommend using a total scale score (index score) across all four factors. Rather, we recommend analyzing the scores of the subscales separately. Unit-weighted mean scores should be computed only for the individual subscales of impulsive behavior. Individual answers should be aggregated to the subscale level only if both items of each subscale were completed. If missing values occur, researchers should use appropriate methods for handling missing data, such as full information maximum likelihood estimation (FIML; [55]), the pseudo-indicator method (PIM; [56]), or multiple imputation [57].

#### Nomological network and sociodemographic variables

Having established factorial validity, the nomological network of I-8 and the associations between the I-8 subscales and the sociodemographic variables were investigated via manifest correlations. Thus, the correlations were lower-bound estimates of the true associations [58]. The correlation coefficients are shown in Table 7. We interpreted the coefficients according to the guidelines recommended by Gignac and Szodorai [59]. Based on over 700 meta-analytically derived correlations in individual differences research, Gignac and Szodorai found that the 25th, 50th, and 75th percentiles corresponded to correlations of *r* = .11, *r* = .19, and *r* = .29. Thus, correlations of .10, .20, and .30 can be interpreted as relatively small, medium, and large, respectively. In the following section, we look at correlations of the I-8 subscales with the constructs outlined in the Materials section above.

**Table 7:** Correlations of I-8 with relevant variables in the UK and German Samples.

	*r* [95% CI]							
	Urgency		Lack of Premeditation		Lack of Perseverance		Sensation Seeking	
	UK	DE	UK	DE	UK	DE	UK	DE
Big Five								
Extraversion	.05 [−.03, .15]	−.02 [−.11, .07]	−.11 [−.20, −.02]	.02 [−.07, .11]	−**.22** [−.30, −.13]	−**.20** [−.28, −.11]	**.27** [.18, .35]	**.29** [.21, .37]
Agreeableness	**−.20** [−.29, −.11]	**−.22** [−.30, −.13]	−.18 [−.27, −.09]	−**.20** [−.28, −.11]	−.15 [−.23, −.06]	−**.27** [−.35, −.18]	−.10 [−.19, −.01]	−.01 [−.10, .08]
Conscientiousness	**−.37** [−.45, −.29]	**−.35** [−.42, −.27]	−**.24** [−.33, −.16]	−**.30** [−.38, −.22]	−**.41** [−.49, −.33]	−**.55** [−.61, −.48]	−.17 [−.26, −.08]	−.01 [−.10, .08]
Emotional Stability	**−.28** [−.37, −.20]	**−.28** [−.36, −.19]	−**.23** [−.31, −.14]	−.17 [−.25, −.08]	−.19 [−.28, −.10]	−**.25** [−.33, −.16]	.12 [.03, .21]	.14 [.05, .22]
Openness	.04 [−.05, .13]	−.05 [−.14, .04]	−.17 [−.25, −.08]	−.12 [−.20, −.03]	−.12 [−.21, −.03]	−.16 [−.24, −.07]	**.27** [.18, .35]	**.22** [.13, .30]
Self-esteem	**−.25** [**−**.33, **−**.16]	**−.29** [**−**.37, **−**.20]	**−**.19 [−.28, −.10]	**−**.19 [**−**.28, **−**.11]	**−.23** [**−**.31, **−**.14]	**−.35** [**−**.43, **−**.27]	.09 [−.00, .18]	.05 [**−**.04, .14]
General self-efficacy	.01 [−.08, .10]	−.09 [−.18, .00]	−**.36** [−.44, −.28]	−**.31** [−.39, −.22]	−**.42** [−.50, −.35]	−**.43** [−.50, −.35]	**.33** [.25, .41]	**.30** [.22, .38]
Locus of control								
Internal	.12 [.03, .21]	−.03 [−.12, .06]	−**.25** [−.33, −.16]	−.18 [−.26, −.09]	−**.36** [−.43, −.28]	−**.43** [−.50, −.35]	**.31** [.23, .39]	**.26** [.18, .34]
External	**.39** [.31, .46]	**.24** [.15, .32]	−.15 [−.24, −.06]	.07 [−.02, .16]	−.16 [−.25, −.07]	.15 [.06, .24]	**.24** [.16, .33]	.05 [**−**.04, .14]
Life satisfaction	−.07 [−.16, .02]	−.11 [−.20, −.02]	−**.20** [−.29, −.12]	−.07 [−.16, .02]	−**.21** [−.30, −.13]	−**.21** [−.30, −.12]	.14 [.05, .22]	.11 [.02, .20]
Risk proneness	**.37** [.29, .45]	**.21** [.12, .29]	.08 [−.01, .17]	.11 [.02, .20]	−.05 [−.14, .04]	−.06 [−.15, .03]	**.73** [.69, .77]	**.73** [.68, .77]
Health	−.12 [−.21, −.03]	−.08 [−.17, .01]	−.09 [−.18, −.00]	−.07 [−.16, .02]	−.14 [−.23, −.05]	−.10 [−.19, −.01]	.11 [.02, .20]	.14 [.05, .22]
Social desirability								
PQ+	−.10 [−.19, −.01]	**−.28** [−.36, −.19]	−**.36** [−.44, −.28]	−**.40** [−.47, −.32]	−**.31** [−.39, −.22]	−**.38** [−.45, −.30]	.13 [.04, .21]	.04 [−.05, .13]
NQ‒	−**.46** [−.53, −.38]	−**.30** [−.38, −.22]	.06 [−.03, .15]	−**.20** [−.29, −.11]	.07 [−.02, .16]	−.18 [−.27, −.10]	−**.34** [−.41, −.25]	−**.20** [−.28, −.11]
Sociodemographic characteristics								
Employment status	−.05 [−.16, .05]	−.10 [−.21, .02]	−.07 [−.18, .03]	−.07 [−.18, .04]	−.10 [−.21, .00]	−.13 [−.23, −.01]	.04 [−.07, .15]	.11 [−.00, .22]
Income	.08 [−.02, .17]	−.06 [−.15, .03]	−.12 [−.22, −.03]	−.11 [−.20, −.02]	−.15 [−.24, −.05]	−.12 [−.21, −.02]	**.22** [.12, .30]	.03 [−.06, .12]
Educational attainment	−.01 [−.10, .08]	−.10 [−.19, −.01]	−.17 [−.26, −.08]	−.14 [−.22, −.05]	−.07 [−.16, .02]	−.04 [−.13, .05]	.06 [−.03, .15]	.05 [−.04, .14]
Age	**−.24** [−.32, −.15]	−.16 [−.25, −.07]	−.01 [−.10, .08]	−.07 [−.16, .02]	.07 [−.02, .16]	−.12 [−.20, −.02]	**−.34** [−.42, −.26]	**−.26** [−.34, −.18]
Sex	−.08 [−.17, .01]	.04 [−.05, .13]	.05 [−.04, .14]	.10 [.01, .19]	−.03 [−.12, .06]	−.02 [−.11, .07]	−.18 [−.26, −.09]	−.09 [−.18, −.00]
Similarity of correlations in UK & DE	.89 [.74, .96]		.79 [.54, .92]		.85 [.64, .94]		.95 [.88, .98]	

*Note*. UK = United Kingdom (*N =* 468; *N*_Left–right self-placement_ = 325; *N*_Employment_ = 339; *N*_Income_ = 431); DE = Germany (*N =* 474; *N*_Left–right self-placement_ = 394; *N*_Employment_ = 309; *N*_Income_ = 449); CI = confidence interval; PQ+ = exaggerating positive qualities; NQ‒ = minimizing negative qualities. Employment status: (1) *unemployed*, (2) *employed*. Sex: (1) *male*, (2) f*emale*. The English version of the Self-Esteem Scale was reverse-coded. We assimilated the direction of the response categories of the English version to the German version of the Self-Esteem Scale. Health was recoded in both nations, so that higher values imply better health. We further recoded NQ‒ so that high scores on both PQ+ and NQ‒ imply stronger social desirability. Medium correlations (*r* ≥ .20) are printed in bold.

The four I-8 subscales showed differential patterns of correlations with variables from the nomological network. As expected, in both nations, Extraversion correlated positively with sensation seeking; Conscientiousness correlated negatively with lack of premeditation and lack of perseverance; and Emotional Stability correlated negatively with urgency (see also [4]). Furthermore, in both nations, Openness was positively related to sensation seeking, which was to be expected due to their overlapping definitions [4]. Interestingly, we found a large negative correlation between Conscientiousness and urgency in both nations (see also [4]).

As expected based on their respective definitions, general self-efficacy correlated negatively with lack of premeditation and lack of perseverance and positively with sensation seeking in both nations (see also [1, 60]). As also expected based on their definitions, internal locus of control was negatively related to lack of premeditation in the UK and to lack of perseverance in both nations; it was positively related to sensation seeking in both nations (see also [4]). Furthermore, we could replicate the findings of Ravert and Donnellan insofar as we observed a small negative association between urgency and life satisfaction in both nations, and a larger negative association between lack of premeditation and life satisfaction in the UK [24]. The overlapping definition of risk proneness and sensation seeking was reflected in their large positive correlation. The negligible correlations of urgency, lack of premeditation, lack of perseverance, and sensation seeking with general health may reflect our earlier acknowledgment that these four factors are related to many psychological disorders, but not to physiological disorders.

The fact that impulsive behavior is deemed socially undesirable was reflected in the large negative correlations of the four I-8 factors with the two key aspects of socially desirable responding—maximizing positive qualities and minimizing negative qualities.

Sociodemographic characteristics have rarely been considered as correlates of impulsivity, which might reflect the fact that the four facets are broadly invariant across the major sociodemographic variables. We found little evidence for differences in the four facets across sociodemographic segments. The only exception was sensation seeking, which was typically found among younger respondents (in both nations) and among respondents with higher income (in the UK). Respondents who scored high on urgency were typically younger. We found no relevant associations between lack of premeditation and perseverance and sociodemographic characteristics.

Overall, the correlations across nations were highly similar, as evidenced by the strong metacorrelations (i.e., the correlation of the correlations from the UK and Germany), which ranged from .79 to .95 (see the bottom row in Table 7). This implies that the nomological networks of all four dimensions were largely equivalent in both countries.

### Measurement invariance across the UK and Germany

We evaluated the comparability of I-8 across the UK and Germany via measurement invariance tests by means of multiple-group CFA [21, 61–63]. We examined measurement invariance in a sequential fashion: First, we investigated configural invariance (same measurement model), then metric invariance (same factor loadings; required for comparing latent (co)variances) and scalar invariance (same item intercepts; required for comparing latent and observed means). We also investigated the invariance of residual variances (equal precision). We further investigated the structural invariance of latent variances (equal latent variances), latent covariances (equal latent covariances), and latent means (equal latent means). Cutoffs for fit indices helped us to evaluate the levels of measurement invariance reached. We applied the cutoffs proposed by Hu and Bentler to assess the absolute magnitude of fit indices [51]. According to Hu and Bentler, CFI should be below .950, RMSEA should be below .060, and SRMR should be below .080 to indicate a well-fitting model. We applied the cutoffs Chen recommended to evaluate the changes in fit indices, comparing a more restricted invariance level to a less restricted one [64]. According to Chen, metric non-invariance is given when CFI changes ≤ −.010 supplemented by changes of ≥ .015 in RMSEA or changes of ≥ .030 in SRMR, comparing the metric to the configural model. Scalar non-invariance (or non-invariance of residual variances) is given when CFI changes ≤ −.010 supplemented by changes of ≥ .015 in RMSEA or changes of ≥ .010 in SRMR, comparing the scalar to the metric model (or the invariance model of residual variances to the scalar model). We apply the latter cutoffs also for evaluating the structural non-invariance of latent variances, latent covariances, and latent means.

Invariance tests were based on the essentially tau-equivalent model identified by setting the loadings of each factor to one and first intercept to zero. Thus, the configural and metric models were equivalent.

Table 8 shows the fit of the different models. The fit indices suggested that the metric model had an acceptable fit to the data, indicating the comparability of the latent variances and covariances across the UK and Germany [65]. However, when the scalar model was tested, the misfit induced by constraining the intercepts was not acceptable relative to the less restricted model.

**Table 8:** Fit of different models testing for invariance.

	Fit indices									Accepted?
Model	χ^2^	*df*	*p*	CFI	Robust CFI	RMSEA	Robust RMSEA	SRMR	BIC	
Metric	41.08	36	.258	.998	.998	.017	.018	.022	18,567	Yes
Scalar	87.71 (Δ50.82)	40 (Δ4)	.000 (Δ.000)	.981 (Δ – .017)	.985 (Δ – .013)	.050 (Δ.033)	.053 (Δ.035)	.031 (Δ.009)	18,591 (Δ24)	No
Partial scalar^a^	65.06 (Δ26.54)	39 (Δ3)	.006 (Δ.000)	.990 (Δ – .008)	.992 (Δ – .006)	.038 (Δ.021)	.039 (Δ.021)	.027 (Δ.005)	18,573 (Δ6)	Yes
Residual variances	102.25 (Δ31.84)	47 (Δ8)	.000 (Δ.000)	.978 (Δ – .012)	.981 (Δ – .011)	.050 (Δ.012)	.054 (Δ.015)	.032 (Δ.005)	18,565 (Δ – 8)	Yes
Latent variances	142.57 (Δ52.26)	51 (Δ4)	.000 (Δ.000)	.964 (Δ – .014)	.969 (Δ – .012)	.062 (Δ.012)	.066 (Δ.012)	.105 (Δ.073)	18,582 (Δ17)	No
Latent covariances	140.58 (Δ38.39)	53 (Δ6)	.000 (Δ.000)	.966 (Δ – .012)	.970 (Δ – .011)	.059 (Δ.009)	.064 (Δ.010)	.081 (Δ.049)	18,569 (Δ4)	No
Latent means	159.36 (Δ63.34)	51 (Δ4)	.006 (Δ.000)	.957 (Δ – .021)	.963 (Δ – .018)	.067 (Δ.017)	.072 (Δ.018)	.066 (Δ.034)	18,603 (Δ39)	No

*Note*. ^a^The intercept with the largest modification index belonged to the first item of the urgency subscale (MI_Urgency1_ = 24.40). RMSEA = root-mean-square error of approximation; CFI = comparative fit index; SRMR = standardized root mean residual; BIC = Bayesian information criterion. The configural invariance model is equivalent to the metric invariance model because the measurement invariance tests are based on the essentially tau-equivalent model.

When the *full* scalar model cannot be accepted, a so-called *partial* scalar model can be tested, thereby allowing non-invariant parameters to be freely estimated [66, 67]. Thus, the partial scalar model has a common-group and a group-specific share of parameters. Non-invariant intercepts can be identified via modification indices (MI), showing the change in the χ^2^ test statistic if the corresponding parameter were released [68]. The intercept with the largest modification index belonged to the first item of the urgency subscale (MI_Urgency1_ = 24.40). Releasing that intercept resulted in a well-fitting and acceptable partial scalar model in both absolute and relative terms. More specifically, all subscales were scalar invariant, except for the urgency subscale, which was only metric invariant. The urgency subscale cannot even be deemed to be partial scalar invariant, as partial scalar invariance implies that at least two intercepts are invariant across groups [66]. The urgency subscale had only one invariant intercept. However, the intercept difference on the urgency subscale was relatively small (τ_Item 1, UK_ ‒ τ_Item 1, DE_ = ‒0.252; standardized difference). We thus tentatively conclude that not only the latent (co)variances, latent means, and observed means for lack of perseverance, lack of premeditation, and sensation seeking but also the ones for urgency can be compared across nations without incurring major bias, although care should be exerted when comparing observed means for urgency across nations (see also [61]).

Further, we tested the invariance of residual variances. The model with equal residual variances for the UK and Germany fit slightly worse than the partial scalar invariance model according to most fit indices (i.e., χ^2^, CFI, RMSEA, SRMR, but not BIC). As only the ΔCFI cutoff was slightly exceeded (i.e., ΔCFI = ‒.012, which should be ΔCFI > −.010) and all other cutoffs were passed, we accepted the model with equal residual variances. When a model with equal residual variances for the UK and Germany fit, the precision across nations is the same [67]. Observed (co)variances can be compared across the UK and Germany without inducing bias [69].

Additionally, we evaluated the structural invariance [62] by setting the latent variances, latent covariances, and latent means equal across the UK and Germany. All these parameters can be compared across groups (as evaluated by the previous measurement invariance tests). We hereby evaluate a substantial question: Do the UK and Germany have equal latent variances, latent covariances, and latent means of the I-8 subscales urgency, lack of premeditation, lack of perseverance, and sensation seeking? To evaluate the substantial question, we restricted either the latent variances, latent covariances, or latent means of the four subscales to be equal across nations (in addition to the residual variances, intercepts, and loadings). Then, we compared the values of fit indices to those of the invariance model up to the level of residual variances. According to Chen’s cutoffs [64], the increase in misfit was substantial for all of these models. Thus, latent variances, latent covariances, and latent means of the I-8 subscales differ across the UK and Germany.

## Discussion and conclusion

The present study aimed to empirically assess the psychometric properties of the Impulsive Behavior Short Scale–8 (I-8) [4]; a scale that measures the four personality facets related to impulsive behavior (i.e., urgency, lack of premeditation, lack of perseverance, and sensation seeking) in a psychometrically valid and comparable manner across the English- and German-language. Our results were based on two heterogeneous quota samples from the UK and Germany. They showed, first, that the English-language I-8 is a reliable and valid instrument for measuring impulsive behavior; and, second, that its psychometric properties are comparable to those of the German-language source instrument. Measurement invariance testing suggested partial scalar invariance and full invariance of residual variances, supporting the comparability of observed (co)variances and latent means across the UK and Germany. I-8 may be used in non-clinical research settings, but it is not suitable for use in individual diagnostics.

Moreover, we could confirm the four-dimensional structure of the I-8 postulated by Kovaleva et al. with the subscales urgency, lack of premeditation, lack of perseverance, and sensation seeking [4]. As expected, these subscales showed the hypothesized differential associations with other psychological constructs, indicating evidence of convergent and divergent validity. Thus, we could replicate and expand Kovaleva et al.’s findings concerning these correlations [4].

However, the scope of our study was limited in four ways. The first two limitations pertain to the scale itself, developed and translated by Kovaleva et al. [4]. The last two limitations pertain to the study design.

First, the items of the three subscales urgency, lack of premeditation, and sensation seeking are worded relatively similarly. The two items of the urgency subscales start with “Sometimes I do things” (Item 1) and “I sometimes do things” (Item 2). Alike, both items of the lack of premeditation subscale start with “I usually” (Items 3 and 4) and the ones of the sensation seeking subscale with “I am” (Items 7 and 8). Thus, parts of the item covariance might result from the similar wording across items for the urgency, lack of premeditation, and sensation seeking subscales. These so-called method biases [70] cannot be separated from the traits in this case, because the trait variance and the method variance are obtained from the same items.

Second, the subscales lack of premeditation and lack of perseverance only contain negatively worded (i.e., reverse coded) items in relation to impulsivity, whereas the subscales urgency and sensation seeking only contain positively worded items. These method biases [70] may inflate the covariance between the subscales lack of premeditation and lack of perseverance as well as urgency and sensation seeking.

Third, the German-language source version of I-8 has evidence of construct validity with the UPPS scale [1] from which it was derived, as well as with well-researched related harmful and risky behaviors, such as self-reported delinquency [4]. In the present sample, we did not validate the English- and German-language I-8 against other existing impulsive behavior scales or other well-researched related constructs. This was because the online survey also served to validate other short scales, and there was no room for specific validation scales besides the project’s core scales. Future research could survey I-8 in combination with related/standard impulsive behavior scales to confirm the assumed construct validity.

Fourth, our validation study was restricted to a UK sample and to respondents in an online survey. Thus, our results may not be generalizable to other English-speaking populations and to individuals without computer skills. Although we do not expect large differences, future research should address these limitations.

Taken together, the results of the present study support the psychometric quality of the English-language version of I-8 and indicate that the scale is excellently suited for cross-national comparisons as it shows highly equal correlations with various extraneous variables from the nomological network and with sociodemographic characteristics. Moreover, except for the non-invariant intercept of the urgency subscale, I-8 showed measurement invariance up to the level of residual variances across the UK and Germany, which further underlines its outstanding suitability for cross-national research involving English-speaking and German-speaking respondents. We thus recommend I-8 for the economic assessment of impulsivity in cross-national survey research, particularly in multi-thematic studies in which assessment time is strictly limited.

## Supporting information

S1 Appendix Answer sheet (English-language version). Impulsive behavior short Scale–8 (I-8).
(PDF)

S2 Appendix Answer sheet (German-language version). Skala impulsives-verhalten–8 (I-8).
(PDF)

S3 Appendix R code for analysis. (PDF)
