# Supplementary material for: The impulsive behavior short scale–8 (I-8): A comprehensive validation of the English-language adaptation
Source: PLoS One. 2022 Sep 6;17(9):e0273801. doi: 10.1371/journal.pone.0273801 (PMC9447926; doi:10.1371/journal.pone.0273801)
Supplement: S1 Appendix — Impulsive behavior short Scale–8 (I-8). (PDF) [file pone.0273801.s001.pdf]

Groskurth K, Nießen D, Rammstedt B, Lechner CM. The Impulsive Behavior Short Scale–8 (I-8): A comprehensive validation of the English-language adaptation. PLOS ONE. 2022.

S1 Appendix: Answer Sheet (English-Language Version)

Impulsive Behavior Short Scale–8 (I-8)

The following statements may apply more or less to you. To what extent do you think each statement applies to you personally?

	does not apply at all	applies a bit	applies somewhat	applies mostly	applies completely
Sometimes I do things impulsively that I should not do.	☐	☐	☐	☐	☐
I sometimes do things to cheer myself up that I later regret.	☐	☐	☐	☐	☐
I usually think carefully before I act.	☐	☐	☐	☐	☐
I usually consider things carefully and logically before I make up my mind.	☐	☐	☐	☐	☐
I always bring to an end what I have started.	☐	☐	☐	☐	☐
I plan my schedule so that I get everything done on time.	☐	☐	☐	☐	☐
I am willing to take risks.	☐	☐	☐	☐	☐
I am happy to take chances.	☐	☐	☐	☐	☐
