# Supplementary material for: The impulsive behavior short scale–8 (I-8): A comprehensive validation of the English-language adaptation
Source: PLoS One. 2022 Sep 6;17(9):e0273801. doi: 10.1371/journal.pone.0273801 (PMC9447926; doi:10.1371/journal.pone.0273801)
Supplement: S2 Appendix — Skala impulsives-verhalten–8 (I-8). (PDF) [file pone.0273801.s002.pdf]

S2 Appendix: Answer Sheet (German-Language Version)

Skala Impulsives-Verhalten–8 (I-8)

Die folgenden Aussagen können mehr oder weniger auf Sie zutreffen. Bitte geben Sie bei jeder Aussage an, inwieweit diese auf Sie persönlich zutrifft.

	trifft gar nicht zu	trifft wenig zu	trifft etwas zu	trifft ziemlich zu	trifft voll und ganz zu
Manchmal tue ich spontan Dinge, die ich besser nicht getan hätte.	☐	☐	☐	☐	☐
Um mich besser zu fühlen, mache ich manchmal Sachen, die ich später bereue.	☐	☐	☐	☐	☐
Ich denke normalerweise genau nach, bevor ich etwas unternehme.	☐	☐	☐	☐	☐
Ich entscheide meist nach sorgfältigem und logischem Überlegen.	☐	☐	☐	☐	☐
Was ich begonnen habe, führe ich auch zu Ende.	☐	☐	☐	☐	☐
Ich teile meine Zeit gut ein, so dass ich Aufgaben rechtzeitig erledigen kann.	☐	☐	☐	☐	☐
Ich bin bereit, Risiken einzugehen.	☐	☐	☐	☐	☐
Ich bin gerne bereit, etwas zu wagen.	☐	☐	☐	☐	☐

Source: Kovaleva et al. [4]
