# Supplementary material for: The impulsive behavior short scale–8 (I-8): A comprehensive validation of the English-language adaptation
Source: PLoS One. 2022 Sep 6;17(9):e0273801. doi: 10.1371/journal.pone.0273801 (PMC9447926; doi:10.1371/journal.pone.0273801)
Supplement: S3 Appendix — (PDF) [file pone.0273801.s003.pdf]

S3 Appendix: R Code for Analysis

```
##########
#Analysis
##########

#Clear workspace (run if desired)
rm(list = ls())

# #List of project directories
# dirs <- list(
#   data = "...",
#   analysis = "...")

#Load required packages
if (!require(psych))  { install.packages("psych") } ; library(psych)
if (!require(lavaan)) { install.packages("lavaan") } ; library(lavaan)
if (!require(car)) { install.packages("car") } ; library(car)
if (!require(semTools)) { install.packages("semTools") } ; library(semTools)

#Load dataset
load(paste0(dirs$data, "I-8.rda"))

#Split dataset between countries
I8_D <- subset(I8, subset=(COUN=="1"))
I8_UK <- subset(I8, subset=(COUN=="2"))

########################################

#Recoding variables

#Recode the premeditation items so that
#higher values point to a higher lack of premeditation/tendency to behave impulsively
I8_UK$PREM1 <- 6-I8_UK$PREM1
I8_UK$PREM2 <- 6-I8_UK$PREM2

I8_D$PREM1 <- 6-I8_D$PREM1
I8_D$PREM2 <- 6-I8_D$PREM2

I8_UK$PREM1rt <- 6-I8_UK$PREM1rt
I8_UK$PREM2rt <- 6-I8_UK$PREM2rt

I8_D$PREM1rt <- 6-I8_D$PREM1rt
I8_D$PREM2rt <- 6-I8_D$PREM2rt
```

```
#... and the same needs to be done for perseverance
I8_UK$PERS1 <- 6-I8_UK$PERS1
I8_UK$PERS2 <- 6-I8_UK$PERS2

I8_D$PERS1 <- 6-I8_D$PERS1
I8_D$PERS2 <- 6-I8_D$PERS2

I8_UK$PERS1rt <- 6-I8_UK$PERS1rt
I8_UK$PERS2rt <- 6-I8_UK$PERS2rt

I8_D$PERS1rt <- 6-I8_D$PERS1rt
I8_D$PERS2rt <- 6-I8_D$PERS2rt

#Furthermore, the English version of the self-esteem scale needs to be recoded
#so that higher values imply more self-esteem
I8_UK$RSES1 <- 5-I8_UK$RSES1
I8_UK$RSES2R <- 5-I8_UK$RSES2R
I8_UK$RSES3 <- 5-I8_UK$RSES3
I8_UK$RSES4 <- 5-I8_UK$RSES4
I8_UK$RSES5R <- 5-I8_UK$RSES5R
I8_UK$RSES6R <- 5-I8_UK$RSES6R
I8_UK$RSES7 <- 5-I8_UK$RSES7
I8_UK$RSES8R <- 5-I8_UK$RSES8R
I8_UK$RSES9R <- 5-I8_UK$RSES9R
I8_UK$RSES10 <- 5-I8_UK$RSES10

#Further, health need to be recoded so that higher values imply better health
I8_UK$HEAL <- 6-I8_UK$HEAL

I8_D$HEAL <- 6-I8_D$HEAL

#Additionally, NQ- must be recoded so that
#higher values imply more socially desirable responding
I8_UK$SDNQ1 <- 6-I8_UK$SDNQ1
I8_UK$SDNQ2 <- 6-I8_UK$SDNQ2
I8_UK$SDNQ3 <- 6-I8_UK$SDNQ3

I8_D$SDNQ1 <- 6-I8_D$SDNQ1
I8_D$SDNQ2 <- 6-I8_D$SDNQ2
I8_D$SDNQ3 <- 6-I8_D$SDNQ3

################################
```

```
#Step 0: Sample Characteristics
################################

##UK

nrow(I8_UK)
describe(I8_UK$AGE)
round(describe(I8_UK$SEX)$mean, 3)

table(I8_UK$QUOT)
round((table(I8_UK$QUOT)/nrow(I8_UK))*100, 1)

edu1 <- subset(I8_UK, QUOT == 1 | QUOT == 2 | QUOT == 3 | QUOT == 10 | QUOT == 11 | QUOT == 12)
edu2 <- subset(I8_UK, QUOT == 4 | QUOT == 5 | QUOT == 6 | QUOT == 13 | QUOT == 14 | QUOT == 15)
edu3 <- subset(I8_UK, QUOT == 7 | QUOT == 8 | QUOT == 9 | QUOT == 16 | QUOT == 17 | QUOT == 18)
round(describe(edu1$QUOT)$n/nrow(I8_UK)*100, 1)
round(describe(edu2$QUOT)$n/nrow(I8_UK)*100, 1)
round(describe(edu3$QUOT)$n/nrow(I8_UK)*100, 1)

##Germany

nrow(I8_D)
describe(I8_D$AGE)
round(describe(I8_D$SEX)$mean, 3)

table(I8_D$QUOT)
round((table(I8_D$QUOT)/nrow(I8_D))*100, 1)

edu1 <- subset(I8_D, QUOT == 1 | QUOT == 2 | QUOT == 3 | QUOT == 10 | QUOT == 11 | QUOT == 12)
edu2 <- subset(I8_D, QUOT == 4 | QUOT == 5 | QUOT == 6 | QUOT == 13 | QUOT == 14 | QUOT == 15)
edu3 <- subset(I8_D, QUOT == 7 | QUOT == 8 | QUOT == 9 | QUOT == 16 | QUOT == 17 | QUOT == 18)
round(describe(edu1$QUOT)$n/nrow(I8_D)*100, 1)
round(describe(edu2$QUOT)$n/nrow(I8_D)*100, 1)
round(describe(edu3$QUOT)$n/nrow(I8_D)*100, 1)


########################################################
```

```
#Step 1: Descriptive Statistics and Reference Values
#############################################################

###Descriptive statistics

##UK

describe(I8_UK$URGE1)
describe(I8_UK$URGE2)
describe(I8_UK$PREM1)
describe(I8_UK$PREM2)
describe(I8_UK$PERS1)
describe(I8_UK$PERS2)
describe(I8_UK$SENS1)
describe(I8_UK$SENS2)

##Germany

describe(I8_D$URGE1)
describe(I8_D$URGE2)
describe(I8_D$PREM1)
describe(I8_D$PREM2)
describe(I8_D$PERS1)
describe(I8_D$PERS2)
describe(I8_D$SENS1)
describe(I8_D$SENS2)

####################################################################################

###Reference values

#Quote 1: male, lower education, 18-29
#Quote 2: male, lower education, 30-49
#Quote 3: male, lower education, 50-69
#Quote 4: male, middle education, 18-29
#Quote 5: male, middle education, 30-49
#Quote 6: male, middle education, 50-69
#Quote 7: male, upper education, 18-29
#Quote 8: male, upper education, 30-49
#Quote 9: male, upper education, 50-69
#Quote 10: female, lower education, 18-29
#Quote 11: female, lower education, 30-49
#Quote 12: female, lower education, 50-69
```

```
#Quote 13: female, middle education, 18-29
#Quote 14: female, middle education, 30-49
#Quote 15: female, middle education, 50-69
#Quote 16: female, upper education, 18-29
#Quote 17: female, upper education, 30-49
#Quote 18: female, upper education, 50-69

##UK

I8_UK$URGE <- (I8_UK$URGE1+I8_UK$URGE2)/2
I8_UK$PREM <- (I8_UK$PREM1+I8_UK$PREM2)/2
I8_UK$PERS <- (I8_UK$PERS1+I8_UK$PERS2)/2
I8_UK$SENS <- (I8_UK$SENS1+I8_UK$SENS2)/2
describe(I8_UK$URGE)
describe(I8_UK$PREM)
describe(I8_UK$PERS)
describe(I8_UK$SENS)

#Urgency
tapply(I8_UK$URGE, I8_UK$SEX, describe)
AGE1_UK <- subset(I8_UK, AGE == 18 | AGE == 19 | AGE == 20 | AGE == 21 | AGE == 22 | AGE == 23 | AGE == 24 | AGE == 25 | AGE == 26
                  | AGE == 27 | AGE == 28 | AGE == 29)
AGE2_UK <- subset(I8_UK, AGE == 30 | AGE == 31 | AGE == 32 | AGE == 33 | AGE == 34 | AGE == 35 | AGE == 36 | AGE == 37 | AGE == 38
                  | AGE == 39 | AGE == 40 | AGE == 41 | AGE == 42 | AGE == 43 | AGE == 44 | AGE == 45 | AGE == 46 | AGE == 47
                  | AGE == 48 | AGE == 49)
AGE3_UK <- subset(I8_UK, AGE == 50 | AGE == 51 | AGE == 52 | AGE == 53 | AGE == 54 | AGE == 55 | AGE == 56 | AGE == 57 | AGE == 58
                  | AGE == 59 | AGE == 60 | AGE == 61 | AGE == 62 | AGE == 63 | AGE == 64 | AGE == 65 | AGE == 66 | AGE == 67
                  | AGE == 68 | AGE == 69)

AGE1_UK$URGE <- (AGE1_UK$URGE1+AGE1_UK$URGE2)/2
describe(AGE1_UK$URGE)

AGE2_UK$URGE <- (AGE2_UK$URGE1+AGE2_UK$URGE2)/2
describe(AGE2_UK$URGE)

AGE3_UK$URGE <- (AGE3_UK$URGE1+AGE3_UK$URGE2)/2
describe(AGE3_UK$URGE)
```

```
#Premeditation
tapply(I8_UK$PREM, I8_UK$SEX, describe)

AGE1_UK$PREM <- (AGE1_UK$PREM1+AGE1_UK$PREM2)/2
describe(AGE1_UK$PREM)

AGE2_UK$PREM <- (AGE2_UK$PREM1+AGE2_UK$PREM2)/2
describe(AGE2_UK$PREM)

AGE3_UK$PREM <- (AGE3_UK$PREM1+AGE3_UK$PREM2)/2
describe(AGE3_UK$PREM)

#Perseverance
tapply(I8_UK$PERS, I8_UK$SEX, describe)

AGE1_UK$PERS <- (AGE1_UK$PERS1+AGE1_UK$PERS2)/2
describe(AGE1_UK$PERS)

AGE2_UK$PERS <- (AGE2_UK$PERS1+AGE2_UK$PERS2)/2
describe(AGE2_UK$PERS)

AGE3_UK$PERS <- (AGE3_UK$PERS1+AGE3_UK$PERS2)/2
describe(AGE3_UK$PERS)

#Sensation seeking
tapply(I8_UK$SENS, I8_UK$SEX, describe)

AGE1_UK$SENS <- (AGE1_UK$SENS1+AGE1_UK$SENS2)/2
describe(AGE1_UK$SENS)

AGE2_UK$SENS <- (AGE2_UK$SENS1+AGE2_UK$SENS2)/2
describe(AGE2_UK$SENS)

AGE3_UK$SENS <- (AGE3_UK$SENS1+AGE3_UK$SENS2)/2
describe(AGE3_UK$SENS)

##Germany

I8_D$URGE <- (I8_D$URGE1+I8_D$URGE2)/2
I8_D$PREM <- (I8_D$PREM1+I8_D$PREM2)/2
I8_D$PERS <- (I8_D$PERS1+I8_D$PERS2)/2
I8_D$SENS <- (I8_D$SENS1+I8_D$SENS2)/2
describe(I8_D$URGE)
```

```
describe(I8_D$PREM)
describe(I8_D$PERS)
describe(I8_D$SENS)

#Urgency
tapply(I8_D$URGE, I8_D$SEX, describe)
AGE1_D <- subset(I8_D, AGE == 18 | AGE == 19 | AGE == 20 | AGE == 21 | AGE == 22 |
AGE == 23 | AGE == 24 | AGE == 25 | AGE == 26
        | AGE == 27 | AGE == 28 | AGE == 29)
AGE2_D <- subset(I8_D, AGE == 30 | AGE == 31 | AGE == 32 | AGE == 33 | AGE == 34 |
AGE == 35 | AGE == 36 | AGE == 37 | AGE == 38
        | AGE == 39 | AGE == 40 | AGE == 41 | AGE == 42 | AGE == 43 | AGE == 44 |
AGE == 45 | AGE == 46 | AGE == 47
        | AGE == 48 | AGE == 49)
AGE3_D <- subset(I8_D, AGE == 50 | AGE == 51 | AGE == 52 | AGE == 53 | AGE == 54 |
AGE == 55 | AGE == 56 | AGE == 57 | AGE == 58
        | AGE == 59 | AGE == 60 | AGE == 61 | AGE == 62 | AGE == 63 | AGE == 64 |
AGE == 65 | AGE == 66 | AGE == 67
        | AGE == 68 | AGE == 69)

AGE1_D$URGE <- (AGE1_D$URGE1+AGE1_D$URGE2)/2
describe(AGE1_D$URGE)

AGE2_D$URGE <- (AGE2_D$URGE1+AGE2_D$URGE2)/2
describe(AGE2_D$URGE)

AGE3_D$URGE <- (AGE3_D$URGE1+AGE3_D$URGE2)/2
describe(AGE3_D$URGE)

#Premeditation
tapply(I8_D$PREM, I8_D$SEX, describe)

AGE1_D$PREM <- (AGE1_D$PREM1+AGE1_D$PREM2)/2
describe(AGE1_D$PREM)

AGE2_D$PREM <- (AGE2_D$PREM1+AGE2_D$PREM2)/2
describe(AGE2_D$PREM)

AGE3_D$PREM <- (AGE3_D$PREM1+AGE3_D$PREM2)/2
describe(AGE3_D$PREM)

#Perseverance
tapply(I8_D$PERS, I8_D$SEX, describe)
```

```
AGE1_D$PERS <- (AGE1_D$PERS1+AGE1_D$PERS2)/2
describe(AGE1_D$PERS)

AGE2_D$PERS <- (AGE2_D$PERS1+AGE2_D$PERS2)/2
describe(AGE2_D$PERS)

AGE3_D$PERS <- (AGE3_D$PERS1+AGE3_D$PERS2)/2
describe(AGE3_D$PERS)

#Sensation seeking
tapply(I8_D$SENS, I8_D$SEX, describe)

AGE1_D$SENS <- (AGE1_D$SENS1+AGE1_D$SENS2)/2
describe(AGE1_D$SENS)

AGE2_D$SENS <- (AGE2_D$SENS1+AGE2_D$SENS2)/2
describe(AGE2_D$SENS)

AGE3_D$SENS <- (AGE3_D$SENS1+AGE3_D$SENS2)/2
describe(AGE3_D$SENS)

#####################
#Step 2: Reliability
#####################

##UK

#McDonald's omega

I8_MM_tau <- 'LV_URGE =~ c(a1)*URGE1 + c(a1)*URGE2
	LV_PREM =~ c(a2)*PREM1 + c(a2)*PREM2
	LV_PERS =~ c(a3)*PERS1 + c(a3)*PERS2
	LV_SENS =~ c(a4)*SENS1 + c(a4)*SENS2

	URGE1+PREM1+PERS1+SENS1 ~ 0*1
	LV_URGE+LV_PREM+LV_PERS+LV_SENS ~ NA*1'

I8_MM.fit_UK <- sem(I8_MM_tau, data = I8_UK, estimator = "mlr", missing = "fiml", std.lv
= FALSE)
semTools::reliability(I8_MM.fit_UK)

##Retest reliability
```

```
I8_UK$URGE <- I8_UK$URGE1+I8_UK$URGE2
I8_UK$PREM <- I8_UK$PREM1+I8_UK$PREM2
I8_UK$PERS <- I8_UK$PERS1+I8_UK$PERS2
I8_UK$SENS <- I8_UK$SENS1+I8_UK$SENS2
I8_UK$URGErt <- I8_UK$URGE1rt+I8_UK$URGE2rt
I8_UK$PREMrt <- I8_UK$PREM1rt+I8_UK$PREM2rt
I8_UK$PERSrt <- I8_UK$PERS1rt+I8_UK$PERS2rt
I8_UK$SENSrt <- I8_UK$SENS1rt+I8_UK$SENS2rt
cor.test(I8_UK$URGE, I8_UK$URGErt, use = "pairwise.complete.obs")
cor.test(I8_UK$PREM, I8_UK$PREMrt, use = "pairwise.complete.obs")
cor.test(I8_UK$PERS, I8_UK$PERSrt, use = "pairwise.complete.obs")
cor.test(I8_UK$SENS, I8_UK$SENSrt, use = "pairwise.complete.obs")

################################################################################

##Germany

#McDonald's omega

I8_MM_tau <- 'LV_URGE =~ c(a1)*URGE1 + c(a1)*URGE2
        LV_PREM =~ c(a2)*PREM1 + c(a2)*PREM2
        LV_PERS =~ c(a3)*PERS1 + c(a3)*PERS2
        LV_SENS =~ c(a4)*SENS1 + c(a4)*SENS2

        URGE1+PREM1+PERS1+SENS1 ~ 0*1
        LV_URGE+LV_PREM+LV_PERS+LV_SENS ~ NA*1
        '
I8_MM.fit_D <- sem(I8_MM_tau, data = I8_D, estimator = "mlr", missing = "fiml", std.lv = FALSE)
semTools::reliability(I8_MM.fit_D)

#Retest reliability

I8_D$URGE <- I8_D$URGE1+I8_D$URGE2
I8_D$PREM <- I8_D$PREM1+I8_D$PREM2
I8_D$PERS <- I8_D$PERS1+I8_D$PERS2
I8_D$SENS <- I8_D$SENS1+I8_D$SENS2
I8_D$URGErt <- I8_D$URGE1rt+I8_D$URGE2rt
I8_D$PREMrt <- I8_D$PREM1rt+I8_D$PREM2rt
I8_D$PERSrt <- I8_D$PERS1rt+I8_D$PERS2rt
I8_D$SENSrt <- I8_D$SENS1rt+I8_D$SENS2rt
cor.test(I8_D$URGE, I8_D$URGErt, use = "pairwise.complete.obs")
```

```
cor.test(I8_D$PREM, I8_D$PREMrt, use = "pairwise.complete.obs")
cor.test(I8_D$PERS, I8_D$PERSrt, use = "pairwise.complete.obs")
cor.test(I8_D$SENS, I8_D$SENSrt, use = "pairwise.complete.obs")

###########################
#Step 3: Measurement Model
###########################

##UK

#Congeneric model
I8_MM_con1 <- 'LV_URGE =~ URGE1 + URGE2
	LV_PREM =~ PREM1 + PREM2
	LV_PERS =~ PERS1 + PERS2
	LV_SENS =~ SENS1 + SENS2

	URGE1+PREM1+PERS1+SENS1 ~ 0*1
	LV_URGE+LV_PREM+LV_PERS+LV_SENS ~ NA*1'

I8_MM.fit <- sem(I8_MM_con1, data = I8_UK, group = "COUN", estimator = "mlr",
missing = "fiml", std.lv = FALSE)
summary(I8_MM.fit, standardized = TRUE, fit.measures = TRUE)

#Essentially tau-equivalent model
I8_MM_tau <- 'LV_URGE =~ c(a1)*URGE1 + c(a1)*URGE2
	LV_PREM =~ c(a2)*PREM1 + c(a2)*PREM2
	LV_PERS =~ c(a3)*PERS1 + c(a3)*PERS2
	LV_SENS =~ c(a4)*SENS1 + c(a4)*SENS2

	URGE1+PREM1+PERS1+SENS1 ~ 0*1
	LV_URGE+LV_PREM+LV_PERS+LV_SENS ~ NA*1'

I8_MM.fit <- sem(I8_MM_tau, data = I8_UK, estimator = "mlr", missing = "fiml", std.lv =
FALSE)
summary(I8_MM.fit, standardized = TRUE, fit.measures = TRUE)

##Germany

#Congeneric model
I8_MM_con1 <- 'LV_URGE =~ URGE1 + URGE2
	LV_PREM =~ PREM1 + PREM2
	LV_PERS =~ PERS1 + PERS2
	LV_SENS =~ SENS1 + SENS2
```

```
	URGE1+PREM1+PERS1+SENS1 ~ 0*1
	LV_URGE+LV_PREM+LV_PERS+LV_SENS ~ NA*1'

I8_MM.fit <- sem(I8_MM_con1, data = I8_D, group = "COUN", estimator = "mlr", missing = "fiml", std.lv = FALSE)
summary(I8_MM.fit, standardized = TRUE, fit.measures = TRUE)

#Essentially tau-equivalent model
I8_MM_tau <- 'LV_URGE =~ c(a1)*URGE1 + c(a1)*URGE2
	LV_PREM =~ c(a2)*PREM1 + c(a2)*PREM2
	LV_PERS =~ c(a3)*PERS1 + c(a3)*PERS2
	LV_SENS =~ c(a4)*SENS1 + c(a4)*SENS2

	URGE1+PREM1+PERS1+SENS1 ~ 0*1
	LV_URGE+LV_PREM+LV_PERS+LV_SENS ~ NA*1'

I8_MM.fit <- sem(I8_MM_tau, data = I8_D, estimator = "mlr", missing = "fiml", std.lv = FALSE)
summary(I8_MM.fit, standardized = TRUE, fit.measures = TRUE)

############################################
#Step 4: Construct and Criterion Validity
############################################

##UK

#Empty vectors to save correlations
UK_URGE_cor <- c()
UK_PREM_cor <- c()
UK_PERS_cor <- c()
UK_SENS_cor <- c()

dimension.matrix <- list(c("EXTR", "AGRE", "CONS", "NEGA", "OPEN", "RSES", "ASKU", "ILOC", "ELOC", "LISA1", "RISK1", "HEAL", "SDPQ", "SDNQ", "EMPL.empl", "INCO", "SCHO", "AGE", "SEX"), c("UK", "DE"))

##########################
#Impulsive behavior (I-8)
##########################
I8_UK$URGE <- I8_UK$URGE1+I8_UK$URGE2
I8_UK$PREM <- I8_UK$PREM1+I8_UK$PREM2
I8_UK$PERS <- I8_UK$PERS1+I8_UK$PERS2
```

```
I8_UK$SENS <- I8_UK$SENS1+I8_UK$SENS2

#####################
#Big Five (BFI-2-XS)
#####################
I8_UK$EXTR <- I8_UK$EXTR1R+I8_UK$EXTR2+I8_UK$EXTR3 #Extraversion
I8_UK$AGRE <- I8_UK$AGRE1+I8_UK$AGRE2R+I8_UK$AGRE3 #Agreeableness
I8_UK$CONS <- I8_UK$CONS1R+I8_UK$CONS2R+I8_UK$CONS3 #Conscientiousness
I8_UK$NEGA <- I8_UK$NEGA1+I8_UK$NEGA2+I8_UK$NEGA3R #Neuroticism
I8_UK$OPEN <- I8_UK$OPEN1+I8_UK$OPEN2R+I8_UK$OPEN3 #Openness

cor.test(I8_UK$URGE, I8_UK$EXTR, use = "pairwise.complete.obs")
UK_URGE_cor <- c(UK_URGE_cor, cor.test(I8_UK$URGE, I8_UK$EXTR, use =
"pairwise.complete.obs")[["estimate"]][["cor"]])
cor.test(I8_UK$PREM, I8_UK$EXTR, use = "pairwise.complete.obs")
UK_PREM_cor <- c(UK_PREM_cor, cor.test(I8_UK$PREM, I8_UK$EXTR, use =
"pairwise.complete.obs")[["estimate"]][["cor"]])
cor.test(I8_UK$PERS, I8_UK$EXTR, use = "pairwise.complete.obs")
UK_PERS_cor <- c(UK_PERS_cor, cor.test(I8_UK$PERS, I8_UK$EXTR, use =
"pairwise.complete.obs")[["estimate"]][["cor"]])
cor.test(I8_UK$SENS, I8_UK$EXTR, use = "pairwise.complete.obs")
UK_SENS_cor <- c(UK_SENS_cor, cor.test(I8_UK$SENS, I8_UK$EXTR, use =
"pairwise.complete.obs")[["estimate"]][["cor"]])

cor.test(I8_UK$URGE, I8_UK$AGRE, use = "pairwise.complete.obs")
UK_URGE_cor <- c(UK_URGE_cor, cor.test(I8_UK$URGE, I8_UK$AGRE, use =
"pairwise.complete.obs")[["estimate"]][["cor"]])
cor.test(I8_UK$PREM, I8_UK$AGRE, use = "pairwise.complete.obs")
UK_PREM_cor <- c(UK_PREM_cor, cor.test(I8_UK$PREM, I8_UK$AGRE, use =
"pairwise.complete.obs")[["estimate"]][["cor"]])
cor.test(I8_UK$PERS, I8_UK$AGRE, use = "pairwise.complete.obs")
UK_PERS_cor <- c(UK_PERS_cor, cor.test(I8_UK$PERS, I8_UK$AGRE, use =
"pairwise.complete.obs")[["estimate"]][["cor"]])
cor.test(I8_UK$SENS, I8_UK$AGRE, use = "pairwise.complete.obs")
UK_SENS_cor <- c(UK_SENS_cor, cor.test(I8_UK$SENS, I8_UK$AGRE, use =
"pairwise.complete.obs")[["estimate"]][["cor"]])

cor.test(I8_UK$URGE, I8_UK$CONS, use = "pairwise.complete.obs")
UK_URGE_cor <- c(UK_URGE_cor, cor.test(I8_UK$URGE, I8_UK$CONS, use =
"pairwise.complete.obs")[["estimate"]][["cor"]])
cor.test(I8_UK$PREM, I8_UK$CONS, use = "pairwise.complete.obs")
UK_PREM_cor <- c(UK_PREM_cor, cor.test(I8_UK$PREM, I8_UK$CONS, use =
"pairwise.complete.obs")[["estimate"]][["cor"]])
```

```
cor.test(I8_UK$PERS, I8_UK$CONS, use = "pairwise.complete.obs")
UK_PERS_cor <- c(UK_PERS_cor, cor.test(I8_UK$PERS, I8_UK$CONS, use =
"pairwise.complete.obs")[["estimate"]][["cor"]])
cor.test(I8_UK$SENS, I8_UK$CONS, use = "pairwise.complete.obs")
UK_SENS_cor <- c(UK_SENS_cor, cor.test(I8_UK$SENS, I8_UK$CONS, use =
"pairwise.complete.obs")[["estimate"]][["cor"]])

cor.test(I8_UK$URGE, I8_UK$NEGA, use = "pairwise.complete.obs")
UK_URGE_cor <- c(UK_URGE_cor, cor.test(I8_UK$URGE, I8_UK$NEGA, use =
"pairwise.complete.obs")[["estimate"]][["cor"]])
cor.test(I8_UK$PREM, I8_UK$NEGA, use = "pairwise.complete.obs")
UK_PREM_cor <- c(UK_PREM_cor, cor.test(I8_UK$PREM, I8_UK$NEGA, use =
"pairwise.complete.obs")[["estimate"]][["cor"]])
cor.test(I8_UK$PERS, I8_UK$NEGA, use = "pairwise.complete.obs")
UK_PERS_cor <- c(UK_PERS_cor, cor.test(I8_UK$PERS, I8_UK$NEGA, use =
"pairwise.complete.obs")[["estimate"]][["cor"]])
cor.test(I8_UK$SENS, I8_UK$NEGA, use = "pairwise.complete.obs")
UK_SENS_cor <- c(UK_SENS_cor, cor.test(I8_UK$SENS, I8_UK$NEGA, use =
"pairwise.complete.obs")[["estimate"]][["cor"]])

cor.test(I8_UK$URGE, I8_UK$OPEN, use = "pairwise.complete.obs")
UK_URGE_cor <- c(UK_URGE_cor, cor.test(I8_UK$URGE, I8_UK$OPEN, use =
"pairwise.complete.obs")[["estimate"]][["cor"]])
cor.test(I8_UK$PREM, I8_UK$OPEN, use = "pairwise.complete.obs")
UK_PREM_cor <- c(UK_PREM_cor, cor.test(I8_UK$PREM, I8_UK$OPEN, use =
"pairwise.complete.obs")[["estimate"]][["cor"]])
cor.test(I8_UK$PERS, I8_UK$OPEN, use = "pairwise.complete.obs")
UK_PERS_cor <- c(UK_PERS_cor, cor.test(I8_UK$PERS, I8_UK$OPEN, use =
"pairwise.complete.obs")[["estimate"]][["cor"]])
cor.test(I8_UK$SENS, I8_UK$OPEN, use = "pairwise.complete.obs")
UK_SENS_cor <- c(UK_SENS_cor, cor.test(I8_UK$SENS, I8_UK$OPEN, use =
"pairwise.complete.obs")[["estimate"]][["cor"]])

####################
#Self-esteem (RSES)
####################
I8_UK$RSES <-
I8_UK$RSES1+I8_UK$RSES2R+I8_UK$RSES3+I8_UK$RSES4+I8_UK$RSES5R+I8_UK
$RSES6R+I8_UK$RSES7+I8_UK$RSES8R+I8_UK$RSES9R+I8_UK$RSES10

cor.test(I8_UK$URGE, I8_UK$RSES, use = "pairwise.complete.obs")
UK_URGE_cor <- c(UK_URGE_cor, cor.test(I8_UK$URGE, I8_UK$OPEN, use =
"pairwise.complete.obs")[["estimate"]][["cor"]])
```

```
cor.test(I8_UK$PREM, I8_UK$RSES, use = "pairwise.complete.obs")
UK_PREM_cor <- c(UK_PREM_cor, cor.test(I8_UK$PREM, I8_UK$OPEN, use =
"pairwise.complete.obs")[["estimate"]][["cor"]])
cor.test(I8_UK$PERS, I8_UK$RSES, use = "pairwise.complete.obs")
UK_PERS_cor <- c(UK_PERS_cor, cor.test(I8_UK$PERS, I8_UK$OPEN, use =
"pairwise.complete.obs")[["estimate"]][["cor"]])
cor.test(I8_UK$SENS, I8_UK$RSES, use = "pairwise.complete.obs")
UK_SENS_cor <- c(UK_SENS_cor, cor.test(I8_UK$SENS, I8_UK$OPEN, use =
"pairwise.complete.obs")[["estimate"]][["cor"]])

################################
#General self-efficacy (GSE-3)
################################
I8_UK$ASKU <- I8_UK$ASKU1+I8_UK$ASKU2+I8_UK$ASKU3

cor.test(I8_UK$URGE, I8_UK$ASKU, use = "pairwise.complete.obs")
UK_URGE_cor <- c(UK_URGE_cor, cor.test(I8_UK$URGE, I8_UK$OPEN, use =
"pairwise.complete.obs")[["estimate"]][["cor"]])
cor.test(I8_UK$PREM, I8_UK$ASKU, use = "pairwise.complete.obs")
UK_PREM_cor <- c(UK_PREM_cor, cor.test(I8_UK$PREM, I8_UK$OPEN, use =
"pairwise.complete.obs")[["estimate"]][["cor"]])
cor.test(I8_UK$PERS, I8_UK$ASKU, use = "pairwise.complete.obs")
UK_PERS_cor <- c(UK_PERS_cor, cor.test(I8_UK$PERS, I8_UK$OPEN, use =
"pairwise.complete.obs")[["estimate"]][["cor"]])
cor.test(I8_UK$SENS, I8_UK$ASKU, use = "pairwise.complete.obs")
UK_SENS_cor <- c(UK_SENS_cor, cor.test(I8_UK$SENS, I8_UK$OPEN, use =
"pairwise.complete.obs")[["estimate"]][["cor"]])

############################################
#Internal-external locus of control (IE-4)
############################################
I8_UK$ILOC <- I8_UK$ILOC1+I8_UK$ILOC2
I8_UK$ELOC <- I8_UK$ELOC1+I8_UK$ELOC2

cor.test(I8_UK$URGE, I8_UK$ILOC, use = "pairwise.complete.obs")
UK_URGE_cor <- c(UK_URGE_cor, cor.test(I8_UK$URGE, I8_UK$OPEN, use =
"pairwise.complete.obs")[["estimate"]][["cor"]])
cor.test(I8_UK$PREM, I8_UK$ILOC, use = "pairwise.complete.obs")
UK_PREM_cor <- c(UK_PREM_cor, cor.test(I8_UK$PREM, I8_UK$OPEN, use =
"pairwise.complete.obs")[["estimate"]][["cor"]])
cor.test(I8_UK$PERS, I8_UK$ILOC, use = "pairwise.complete.obs")
UK_PERS_cor <- c(UK_PERS_cor, cor.test(I8_UK$PERS, I8_UK$OPEN, use =
"pairwise.complete.obs")[["estimate"]][["cor"]])
```

```
cor.test(I8_UK$SENS, I8_UK$ILOC, use = "pairwise.complete.obs")
UK_SENS_cor <- c(UK_SENS_cor, cor.test(I8_UK$SENS, I8_UK$OPEN, use =
"pairwise.complete.obs")[["estimate"]][["cor"]])

cor.test(I8_UK$URGE, I8_UK$ELOC, use = "pairwise.complete.obs")
UK_URGE_cor <- c(UK_URGE_cor, cor.test(I8_UK$URGE, I8_UK$OPEN, use =
"pairwise.complete.obs")[["estimate"]][["cor"]])
cor.test(I8_UK$PREM, I8_UK$ELOC, use = "pairwise.complete.obs")
UK_PREM_cor <- c(UK_PREM_cor, cor.test(I8_UK$PREM, I8_UK$OPEN, use =
"pairwise.complete.obs")[["estimate"]][["cor"]])
cor.test(I8_UK$PERS, I8_UK$ELOC, use = "pairwise.complete.obs")
UK_PERS_cor <- c(UK_PERS_cor, cor.test(I8_UK$PERS, I8_UK$OPEN, use =
"pairwise.complete.obs")[["estimate"]][["cor"]])
cor.test(I8_UK$SENS, I8_UK$ELOC, use = "pairwise.complete.obs")
UK_SENS_cor <- c(UK_SENS_cor, cor.test(I8_UK$SENS, I8_UK$OPEN, use =
"pairwise.complete.obs")[["estimate"]][["cor"]])

###################################
#General life satisfaction (L-1)
###################################
cor.test(I8_UK$URGE, I8_UK$LISA1, use = "pairwise.complete.obs")
UK_URGE_cor <- c(UK_URGE_cor, cor.test(I8_UK$URGE, I8_UK$LISA1, use =
"pairwise.complete.obs")[["estimate"]][["cor"]])
cor.test(I8_UK$PREM, I8_UK$LISA1, use = "pairwise.complete.obs")
UK_PREM_cor <- c(UK_PREM_cor, cor.test(I8_UK$PREM, I8_UK$LISA1, use =
"pairwise.complete.obs")[["estimate"]][["cor"]])
cor.test(I8_UK$PERS, I8_UK$LISA1, use = "pairwise.complete.obs")
UK_PERS_cor <- c(UK_PERS_cor, cor.test(I8_UK$PERS, I8_UK$LISA1, use =
"pairwise.complete.obs")[["estimate"]][["cor"]])
cor.test(I8_UK$SENS, I8_UK$LISA1, use = "pairwise.complete.obs")
UK_SENS_cor <- c(UK_SENS_cor, cor.test(I8_UK$SENS, I8_UK$LISA1, use =
"pairwise.complete.obs")[["estimate"]][["cor"]])

######################
#Risk Proneness (R-1)
######################
cor.test(I8_UK$URGE, I8_UK$RISK1, use = "pairwise.complete.obs")
UK_URGE_cor <- c(UK_URGE_cor, cor.test(I8_UK$URGE, I8_UK$RISK1, use =
"pairwise.complete.obs")[["estimate"]][["cor"]])
cor.test(I8_UK$PREM, I8_UK$RISK1, use = "pairwise.complete.obs")
UK_PREM_cor <- c(UK_PREM_cor, cor.test(I8_UK$PREM, I8_UK$RISK1, use =
"pairwise.complete.obs")[["estimate"]][["cor"]])
cor.test(I8_UK$PERS, I8_UK$RISK1, use = "pairwise.complete.obs")
```

```
UK_PERS_cor <- c(UK_PERS_cor, cor.test(I8_UK$PERS, I8_UK$RISK1, use =
"pairwise.complete.obs")[["estimate"]][["cor"]])
cor.test(I8_UK$SENS, I8_UK$RISK1, use = "pairwise.complete.obs")
UK_SENS_cor <- c(UK_SENS_cor, cor.test(I8_UK$SENS, I8_UK$RISK1, use =
"pairwise.complete.obs")[["estimate"]][["cor"]])

########
#Health
########
cor.test(I8_UK$URGE, I8_UK$HEAL, use = "pairwise.complete.obs")
UK_URGE_cor <- c(UK_URGE_cor, cor.test(I8_UK$URGE, I8_UK$HEAL, use =
"pairwise.complete.obs")[["estimate"]][["cor"]])
cor.test(I8_UK$PREM, I8_UK$HEAL, use = "pairwise.complete.obs")
UK_PREM_cor <- c(UK_PREM_cor, cor.test(I8_UK$PREM, I8_UK$HEAL, use =
"pairwise.complete.obs")[["estimate"]][["cor"]])
cor.test(I8_UK$PERS, I8_UK$HEAL, use = "pairwise.complete.obs")
UK_PERS_cor <- c(UK_PERS_cor, cor.test(I8_UK$PERS, I8_UK$HEAL, use =
"pairwise.complete.obs")[["estimate"]][["cor"]])
cor.test(I8_UK$SENS, I8_UK$HEAL, use = "pairwise.complete.obs")
UK_SENS_cor <- c(UK_SENS_cor, cor.test(I8_UK$SENS, I8_UK$HEAL, use =
"pairwise.complete.obs")[["estimate"]][["cor"]])

##############################
#Social desirability (KSE-G)
##############################
I8_UK$SDPQ <- I8_UK$SDPQ1+I8_UK$SDPQ2+I8_UK$SDPQ3
I8_UK$SDNQ <- I8_UK$SDNQ1+I8_UK$SDNQ2+I8_UK$SDNQ3

cor.test(I8_UK$URGE, I8_UK$SDPQ, use = "pairwise.complete.obs")
UK_URGE_cor <- c(UK_URGE_cor, cor.test(I8_UK$URGE, I8_UK$SDPQ, use =
"pairwise.complete.obs")[["estimate"]][["cor"]])
cor.test(I8_UK$PREM, I8_UK$SDPQ, use = "pairwise.complete.obs")
UK_PREM_cor <- c(UK_PREM_cor, cor.test(I8_UK$PREM, I8_UK$SDPQ, use =
"pairwise.complete.obs")[["estimate"]][["cor"]])
cor.test(I8_UK$PERS, I8_UK$SDPQ, use = "pairwise.complete.obs")
UK_PERS_cor <- c(UK_PERS_cor, cor.test(I8_UK$PERS, I8_UK$SDPQ, use =
"pairwise.complete.obs")[["estimate"]][["cor"]])
cor.test(I8_UK$SENS, I8_UK$SDPQ, use = "pairwise.complete.obs")
UK_SENS_cor <- c(UK_SENS_cor, cor.test(I8_UK$SENS, I8_UK$SDPQ, use =
"pairwise.complete.obs")[["estimate"]][["cor"]])

cor.test(I8_UK$URGE, I8_UK$SDNQ, use = "pairwise.complete.obs")
```

```
UK_URGE_cor <- c(UK_URGE_cor, cor.test(I8_UK$URGE, I8_UK$SDNQ, use =
"pairwise.complete.obs")[["estimate"]][["cor"]])
cor.test(I8_UK$PREM, I8_UK$SDNQ, use = "pairwise.complete.obs")
UK_PREM_cor <- c(UK_PREM_cor, cor.test(I8_UK$PREM, I8_UK$SDNQ, use =
"pairwise.complete.obs")[["estimate"]][["cor"]])
cor.test(I8_UK$PERS, I8_UK$SDNQ, use = "pairwise.complete.obs")
UK_PERS_cor <- c(UK_PERS_cor, cor.test(I8_UK$PERS, I8_UK$SDNQ, use =
"pairwise.complete.obs")[["estimate"]][["cor"]])
cor.test(I8_UK$SENS, I8_UK$SDNQ, use = "pairwise.complete.obs")
UK_SENS_cor <- c(UK_SENS_cor, cor.test(I8_UK$SENS, I8_UK$SDNQ, use =
"pairwise.complete.obs")[["estimate"]][["cor"]])

###################
#Employment status
###################
#1) employed
#2) self-employed
#3) out of work and looking for work
#4) out of work but not currently looking for work
#5) doing housework
#6) pupil/student
#7) apprentice/internship
#8) retired
#[9) none of what is mentioned above]
describe(I8_UK$EMPL)
#unemployed vs. employed
I8_UK$EMPL.empl <- recode(I8_UK$EMPL, "3:4 = 1; 1:2 = 2; else = NA")
describe(I8_UK$EMPL.empl)

cor.test(I8_UK$URGE, I8_UK$EMPL.empl, use = "pairwise.complete.obs")
UK_URGE_cor <- c(UK_URGE_cor, cor.test(I8_UK$URGE, I8_UK$EMPL.empl, use =
"pairwise.complete.obs")[["estimate"]][["cor"]])
cor.test(I8_UK$PREM, I8_UK$EMPL.empl, use = "pairwise.complete.obs")
UK_PREM_cor <- c(UK_PREM_cor, cor.test(I8_UK$PREM, I8_UK$EMPL.empl, use =
"pairwise.complete.obs")[["estimate"]][["cor"]])
cor.test(I8_UK$PERS, I8_UK$EMPL.empl, use = "pairwise.complete.obs")
UK_PERS_cor <- c(UK_PERS_cor, cor.test(I8_UK$PERS, I8_UK$EMPL.empl, use =
"pairwise.complete.obs")[["estimate"]][["cor"]])
cor.test(I8_UK$SENS, I8_UK$EMPL.empl, use = "pairwise.complete.obs")
UK_SENS_cor <- c(UK_SENS_cor, cor.test(I8_UK$SENS, I8_UK$EMPL.empl, use =
"pairwise.complete.obs")[["estimate"]][["cor"]])

########
```

```
#Income
########
cor.test(I8_UK$URGE, I8_UK$INCO, use = "pairwise.complete.obs")
UK_URGE_cor <- c(UK_URGE_cor, cor.test(I8_UK$URGE, I8_UK$INCO, use =
"pairwise.complete.obs")[["estimate"]][["cor"]])
cor.test(I8_UK$PREM, I8_UK$INCO, use = "pairwise.complete.obs")
UK_PREM_cor <- c(UK_PREM_cor, cor.test(I8_UK$PREM, I8_UK$INCO, use =
"pairwise.complete.obs")[["estimate"]][["cor"]])
cor.test(I8_UK$PERS, I8_UK$INCO, use = "pairwise.complete.obs")
UK_PERS_cor <- c(UK_PERS_cor, cor.test(I8_UK$PERS, I8_UK$INCO, use =
"pairwise.complete.obs")[["estimate"]][["cor"]])
cor.test(I8_UK$SENS, I8_UK$INCO, use = "pairwise.complete.obs")
UK_SENS_cor <- c(UK_SENS_cor, cor.test(I8_UK$SENS, I8_UK$INCO, use =
"pairwise.complete.obs")[["estimate"]][["cor"]])

########################
#Educational attainment
########################
cor.test(I8_UK$URGE, I8_UK$SCHO, use = "pairwise.complete.obs")
UK_URGE_cor <- c(UK_URGE_cor, cor.test(I8_UK$URGE, I8_UK$SCHO, use =
"pairwise.complete.obs")[["estimate"]][["cor"]])
cor.test(I8_UK$PREM, I8_UK$SCHO, use = "pairwise.complete.obs")
UK_PREM_cor <- c(UK_PREM_cor, cor.test(I8_UK$PREM, I8_UK$SCHO, use =
"pairwise.complete.obs")[["estimate"]][["cor"]])
cor.test(I8_UK$PERS, I8_UK$SCHO, use = "pairwise.complete.obs")
UK_PERS_cor <- c(UK_PERS_cor, cor.test(I8_UK$PERS, I8_UK$SCHO, use =
"pairwise.complete.obs")[["estimate"]][["cor"]])
cor.test(I8_UK$SENS, I8_UK$SCHO, use = "pairwise.complete.obs")
UK_SENS_cor <- c(UK_SENS_cor, cor.test(I8_UK$SENS, I8_UK$SCHO, use =
"pairwise.complete.obs")[["estimate"]][["cor"]])

#####
#Age
#####
cor.test(I8_UK$URGE, I8_UK$AGE, use = "pairwise.complete.obs")
UK_URGE_cor <- c(UK_URGE_cor, cor.test(I8_UK$URGE, I8_UK$AGE, use =
"pairwise.complete.obs")[["estimate"]][["cor"]])
cor.test(I8_UK$PREM, I8_UK$AGE, use = "pairwise.complete.obs")
UK_PREM_cor <- c(UK_PREM_cor, cor.test(I8_UK$PREM, I8_UK$AGE, use =
"pairwise.complete.obs")[["estimate"]][["cor"]])
cor.test(I8_UK$PERS, I8_UK$AGE, use = "pairwise.complete.obs")
UK_PERS_cor <- c(UK_PERS_cor, cor.test(I8_UK$PERS, I8_UK$AGE, use =
"pairwise.complete.obs")[["estimate"]][["cor"]])
```

```
cor.test(I8_UK$SENS, I8_UK$AGE, use = "pairwise.complete.obs")
UK_SENS_cor <- c(UK_SENS_cor, cor.test(I8_UK$SENS, I8_UK$AGE, use =
"pairwise.complete.obs")[["estimate"]][["cor"]])

#####
#Sex
#####
cor.test(I8_UK$URGE, I8_UK$SEX, use = "pairwise.complete.obs")
UK_URGE_cor <- c(UK_URGE_cor, cor.test(I8_UK$URGE, I8_UK$SEX, use =
"pairwise.complete.obs")[["estimate"]][["cor"]])
cor.test(I8_UK$PREM, I8_UK$SEX, use = "pairwise.complete.obs")
UK_PREM_cor <- c(UK_PREM_cor, cor.test(I8_UK$PREM, I8_UK$SEX, use =
"pairwise.complete.obs")[["estimate"]][["cor"]])
cor.test(I8_UK$PERS, I8_UK$SEX, use = "pairwise.complete.obs")
UK_PERS_cor <- c(UK_PERS_cor, cor.test(I8_UK$PERS, I8_UK$SEX, use =
"pairwise.complete.obs")[["estimate"]][["cor"]])
cor.test(I8_UK$SENS, I8_UK$SEX, use = "pairwise.complete.obs")
UK_SENS_cor <- c(UK_SENS_cor, cor.test(I8_UK$SENS, I8_UK$SEX, use =
"pairwise.complete.obs")[["estimate"]][["cor"]])

##############################################################################

##Germany

#Empty vectors to save correlations
DE_URGE_cor <- c()
DE_PREM_cor <- c()
DE_PERS_cor <- c()
DE_SENS_cor <- c()

dimension.matrix <- list(c("EXTR", "AGRE", "CONS", "NEGA", "OPEN", "RSES",
"ASKU", "ILOC", "ELOC", "LISA1", "RISK1", "HEAL", "SDPQ", "SDNQ", "EMPL.empl",
"INCO", "SCHO", "AGE", "SEX"), c("UK", "DE"))

###########################
#Impulsive behavior (I-8)
###########################
I8_D$URGE <- I8_D$URGE1+I8_D$URGE2
I8_D$PREM <- I8_D$PREM1+I8_D$PREM2
I8_D$PERS <- I8_D$PERS1+I8_D$PERS2
I8_D$SENS <- I8_D$SENS1+I8_D$SENS2

####################
```

```
#Big Five (BFI-2-XS)
#####################
I8_D$EXTR <- I8_D$EXTR1R+I8_D$EXTR2+I8_D$EXTR3 #Extraversion
I8_D$AGRE <- I8_D$AGRE1+I8_D$AGRE2R+I8_D$AGRE3 #Agreeableness
I8_D$CONS <- I8_D$CONS1R+I8_D$CONS2R+I8_D$CONS3 #Conscientiousness
I8_D$NEGA <- I8_D$NEGA1+I8_D$NEGA2+I8_D$NEGA3R #Neuroticism
I8_D$OPEN <- I8_D$OPEN1+I8_D$OPEN2R+I8_D$OPEN3 #Openness

cor.test(I8_D$URGE, I8_D$EXTR, use = "pairwise.complete.obs")
DE_URGE_cor <- c(DE_URGE_cor, cor.test(I8_D$URGE, I8_D$EXTR, use =
"pairwise.complete.obs")[["estimate"]][["cor"]])
cor.test(I8_D$PREM, I8_D$EXTR, use = "pairwise.complete.obs")
DE_PREM_cor <- c(DE_PREM_cor, cor.test(I8_D$PREM, I8_D$EXTR, use =
"pairwise.complete.obs")[["estimate"]][["cor"]])
cor.test(I8_D$PERS, I8_D$EXTR, use = "pairwise.complete.obs")
DE_PERS_cor <- c(DE_PERS_cor, cor.test(I8_D$PERS, I8_D$EXTR, use =
"pairwise.complete.obs")[["estimate"]][["cor"]])
cor.test(I8_D$SENS, I8_D$EXTR, use = "pairwise.complete.obs")
DE_SENS_cor <- c(DE_SENS_cor, cor.test(I8_D$SENS, I8_D$EXTR, use =
"pairwise.complete.obs")[["estimate"]][["cor"]])

cor.test(I8_D$URGE, I8_D$AGRE, use = "pairwise.complete.obs")
DE_URGE_cor <- c(DE_URGE_cor, cor.test(I8_D$URGE, I8_D$AGRE, use =
"pairwise.complete.obs")[["estimate"]][["cor"]])
cor.test(I8_D$PREM, I8_D$AGRE, use = "pairwise.complete.obs")
DE_PREM_cor <- c(DE_PREM_cor, cor.test(I8_D$PREM, I8_D$AGRE, use =
"pairwise.complete.obs")[["estimate"]][["cor"]])
cor.test(I8_D$PERS, I8_D$AGRE, use = "pairwise.complete.obs")
DE_PERS_cor <- c(DE_PERS_cor, cor.test(I8_D$PERS, I8_D$AGRE, use =
"pairwise.complete.obs")[["estimate"]][["cor"]])
cor.test(I8_D$SENS, I8_D$AGRE, use = "pairwise.complete.obs")
DE_SENS_cor <- c(DE_SENS_cor, cor.test(I8_D$SENS, I8_D$AGRE, use =
"pairwise.complete.obs")[["estimate"]][["cor"]])

cor.test(I8_D$URGE, I8_D$CONS, use = "pairwise.complete.obs")
DE_URGE_cor <- c(DE_URGE_cor, cor.test(I8_D$URGE, I8_D$CONS, use =
"pairwise.complete.obs")[["estimate"]][["cor"]])
cor.test(I8_D$PREM, I8_D$CONS, use = "pairwise.complete.obs")
DE_PREM_cor <- c(DE_PREM_cor, cor.test(I8_D$PREM, I8_D$CONS, use =
"pairwise.complete.obs")[["estimate"]][["cor"]])
cor.test(I8_D$PERS, I8_D$CONS, use = "pairwise.complete.obs")
DE_PERS_cor <- c(DE_PERS_cor, cor.test(I8_D$PERS, I8_D$CONS, use =
"pairwise.complete.obs")[["estimate"]][["cor"]])
```

```
cor.test(I8_D$SENS, I8_D$CONS, use = "pairwise.complete.obs")
DE_SENS_cor <- c(DE_SENS_cor, cor.test(I8_D$SENS, I8_D$CONS, use =
"pairwise.complete.obs")[["estimate"]][["cor"]])

cor.test(I8_D$URGE, I8_D$NEGA, use = "pairwise.complete.obs")
DE_URGE_cor <- c(DE_URGE_cor, cor.test(I8_D$URGE, I8_D$NEGA, use =
"pairwise.complete.obs")[["estimate"]][["cor"]])
cor.test(I8_D$PREM, I8_D$NEGA, use = "pairwise.complete.obs")
DE_PREM_cor <- c(DE_PREM_cor, cor.test(I8_D$PREM, I8_D$NEGA, use =
"pairwise.complete.obs")[["estimate"]][["cor"]])
cor.test(I8_D$PERS, I8_D$NEGA, use = "pairwise.complete.obs")
DE_PERS_cor <- c(DE_PERS_cor, cor.test(I8_D$PERS, I8_D$NEGA, use =
"pairwise.complete.obs")[["estimate"]][["cor"]])
cor.test(I8_D$SENS, I8_D$NEGA, use = "pairwise.complete.obs")
DE_SENS_cor <- c(DE_SENS_cor, cor.test(I8_D$SENS, I8_D$NEGA, use =
"pairwise.complete.obs")[["estimate"]][["cor"]])

cor.test(I8_D$URGE, I8_D$OPEN, use = "pairwise.complete.obs")
DE_URGE_cor <- c(DE_URGE_cor, cor.test(I8_D$URGE, I8_D$OPEN, use =
"pairwise.complete.obs")[["estimate"]][["cor"]])
cor.test(I8_D$PREM, I8_D$OPEN, use = "pairwise.complete.obs")
DE_PREM_cor <- c(DE_PREM_cor, cor.test(I8_D$PREM, I8_D$OPEN, use =
"pairwise.complete.obs")[["estimate"]][["cor"]])
cor.test(I8_D$PERS, I8_D$OPEN, use = "pairwise.complete.obs")
DE_PERS_cor <- c(DE_PERS_cor, cor.test(I8_D$PERS, I8_D$OPEN, use =
"pairwise.complete.obs")[["estimate"]][["cor"]])
cor.test(I8_D$SENS, I8_D$OPEN, use = "pairwise.complete.obs")
DE_SENS_cor <- c(DE_SENS_cor, cor.test(I8_D$SENS, I8_D$OPEN, use =
"pairwise.complete.obs")[["estimate"]][["cor"]])

####################
#Self-esteem (RSES)
####################
I8_D$RSES <-
I8_D$RSES1+I8_D$RSES2R+I8_D$RSES3+I8_D$RSES4+I8_D$RSES5R+I8_D$RSES6R
+I8_D$RSES7+I8_D$RSES8R+I8_D$RSES9R+I8_D$RSES10

cor.test(I8_D$URGE, I8_D$RSES, use = "pairwise.complete.obs")
DE_URGE_cor <- c(DE_URGE_cor, cor.test(I8_D$URGE, I8_D$OPEN, use =
"pairwise.complete.obs")[["estimate"]][["cor"]])
cor.test(I8_D$PREM, I8_D$RSES, use = "pairwise.complete.obs")
DE_PREM_cor <- c(DE_PREM_cor, cor.test(I8_D$PREM, I8_D$OPEN, use =
"pairwise.complete.obs")[["estimate"]][["cor"]])
```

```
cor.test(I8_D$PERS, I8_D$RSES, use = "pairwise.complete.obs")
DE_PERS_cor <- c(DE_PERS_cor, cor.test(I8_D$PERS, I8_D$OPEN, use =
"pairwise.complete.obs")[["estimate"]][["cor"]])
cor.test(I8_D$SENS, I8_D$RSES, use = "pairwise.complete.obs")
DE_SENS_cor <- c(DE_SENS_cor, cor.test(I8_D$SENS, I8_D$OPEN, use =
"pairwise.complete.obs")[["estimate"]][["cor"]])

################################
#General self-efficacy (ASKU)
################################
I8_D$ASKU <- I8_D$ASKU1+I8_D$ASKU2+I8_D$ASKU3

cor.test(I8_D$URGE, I8_D$ASKU, use = "pairwise.complete.obs")
DE_URGE_cor <- c(DE_URGE_cor, cor.test(I8_D$URGE, I8_D$OPEN, use =
"pairwise.complete.obs")[["estimate"]][["cor"]])
cor.test(I8_D$PREM, I8_D$ASKU, use = "pairwise.complete.obs")
DE_PREM_cor <- c(DE_PREM_cor, cor.test(I8_D$PREM, I8_D$OPEN, use =
"pairwise.complete.obs")[["estimate"]][["cor"]])
cor.test(I8_D$PERS, I8_D$ASKU, use = "pairwise.complete.obs")
DE_PERS_cor <- c(DE_PERS_cor, cor.test(I8_D$PERS, I8_D$OPEN, use =
"pairwise.complete.obs")[["estimate"]][["cor"]])
cor.test(I8_D$SENS, I8_D$ASKU, use = "pairwise.complete.obs")
DE_SENS_cor <- c(DE_SENS_cor, cor.test(I8_D$SENS, I8_D$OPEN, use =
"pairwise.complete.obs")[["estimate"]][["cor"]])

##############################################
#Internal-external locus of control (IE-4)
##############################################
I8_D$ILOC <- I8_D$ILOC1+I8_D$ILOC2
I8_D$ELOC <- I8_D$ELOC1+I8_D$ELOC2

cor.test(I8_D$URGE, I8_D$ILOC, use = "pairwise.complete.obs")
DE_URGE_cor <- c(DE_URGE_cor, cor.test(I8_D$URGE, I8_D$OPEN, use =
"pairwise.complete.obs")[["estimate"]][["cor"]])
cor.test(I8_D$PREM, I8_D$ILOC, use = "pairwise.complete.obs")
DE_PREM_cor <- c(DE_PREM_cor, cor.test(I8_D$PREM, I8_D$OPEN, use =
"pairwise.complete.obs")[["estimate"]][["cor"]])
cor.test(I8_D$PERS, I8_D$ILOC, use = "pairwise.complete.obs")
DE_PERS_cor <- c(DE_PERS_cor, cor.test(I8_D$PERS, I8_D$OPEN, use =
"pairwise.complete.obs")[["estimate"]][["cor"]])
cor.test(I8_D$SENS, I8_D$ILOC, use = "pairwise.complete.obs")
DE_SENS_cor <- c(DE_SENS_cor, cor.test(I8_D$SENS, I8_D$OPEN, use =
"pairwise.complete.obs")[["estimate"]][["cor"]])
```

```
cor.test(I8_D$URGE, I8_D$ELOC, use = "pairwise.complete.obs")
DE_URGE_cor <- c(DE_URGE_cor, cor.test(I8_D$URGE, I8_D$OPEN, use =
"pairwise.complete.obs")[["estimate"]][["cor"]])
cor.test(I8_D$PREM, I8_D$ELOC, use = "pairwise.complete.obs")
DE_PREM_cor <- c(DE_PREM_cor, cor.test(I8_D$PREM, I8_D$OPEN, use =
"pairwise.complete.obs")[["estimate"]][["cor"]])
cor.test(I8_D$PERS, I8_D$ELOC, use = "pairwise.complete.obs")
DE_PERS_cor <- c(DE_PERS_cor, cor.test(I8_D$PERS, I8_D$OPEN, use =
"pairwise.complete.obs")[["estimate"]][["cor"]])
cor.test(I8_D$SENS, I8_D$ELOC, use = "pairwise.complete.obs")
DE_SENS_cor <- c(DE_SENS_cor, cor.test(I8_D$SENS, I8_D$OPEN, use =
"pairwise.complete.obs")[["estimate"]][["cor"]])

###################################
#General life satisfaction (L-1)
###################################
cor.test(I8_D$URGE, I8_D$LISA1, use = "pairwise.complete.obs")
DE_URGE_cor <- c(DE_URGE_cor, cor.test(I8_D$URGE, I8_D$LISA1, use =
"pairwise.complete.obs")[["estimate"]][["cor"]])
cor.test(I8_D$PREM, I8_D$LISA1, use = "pairwise.complete.obs")
DE_PREM_cor <- c(DE_PREM_cor, cor.test(I8_D$PREM, I8_D$LISA1, use =
"pairwise.complete.obs")[["estimate"]][["cor"]])
cor.test(I8_D$PERS, I8_D$LISA1, use = "pairwise.complete.obs")
DE_PERS_cor <- c(DE_PERS_cor, cor.test(I8_D$PERS, I8_D$LISA1, use =
"pairwise.complete.obs")[["estimate"]][["cor"]])
cor.test(I8_D$SENS, I8_D$LISA1, use = "pairwise.complete.obs")
DE_SENS_cor <- c(DE_SENS_cor, cor.test(I8_D$SENS, I8_D$LISA1, use =
"pairwise.complete.obs")[["estimate"]][["cor"]])

######################
#Risk proneness (R-1)
######################
cor.test(I8_D$URGE, I8_D$RISK1, use = "pairwise.complete.obs")
DE_URGE_cor <- c(DE_URGE_cor, cor.test(I8_D$URGE, I8_D$RISK1, use =
"pairwise.complete.obs")[["estimate"]][["cor"]])
cor.test(I8_D$PREM, I8_D$RISK1, use = "pairwise.complete.obs")
DE_PREM_cor <- c(DE_PREM_cor, cor.test(I8_D$PREM, I8_D$RISK1, use =
"pairwise.complete.obs")[["estimate"]][["cor"]])
cor.test(I8_D$PERS, I8_D$RISK1, use = "pairwise.complete.obs")
DE_PERS_cor <- c(DE_PERS_cor, cor.test(I8_D$PERS, I8_D$RISK1, use =
"pairwise.complete.obs")[["estimate"]][["cor"]])
cor.test(I8_D$SENS, I8_D$RISK1, use = "pairwise.complete.obs")
```

```
DE_SENS_cor <- c(DE_SENS_cor, cor.test(I8_D$SENS, I8_D$RISK1, use =
"pairwise.complete.obs")[["estimate"]][["cor"]])

########
#Health
########
cor.test(I8_D$URGE, I8_D$HEAL, use = "pairwise.complete.obs")
DE_URGE_cor <- c(DE_URGE_cor, cor.test(I8_D$URGE, I8_D$HEAL, use =
"pairwise.complete.obs")[["estimate"]][["cor"]])
cor.test(I8_D$PREM, I8_D$HEAL, use = "pairwise.complete.obs")
DE_PREM_cor <- c(DE_PREM_cor, cor.test(I8_D$PREM, I8_D$HEAL, use =
"pairwise.complete.obs")[["estimate"]][["cor"]])
cor.test(I8_D$PERS, I8_D$HEAL, use = "pairwise.complete.obs")
DE_PERS_cor <- c(DE_PERS_cor, cor.test(I8_D$PERS, I8_D$HEAL, use =
"pairwise.complete.obs")[["estimate"]][["cor"]])
cor.test(I8_D$SENS, I8_D$HEAL, use = "pairwise.complete.obs")
DE_SENS_cor <- c(DE_SENS_cor, cor.test(I8_D$SENS, I8_D$HEAL, use =
"pairwise.complete.obs")[["estimate"]][["cor"]])

##############################
#Social desirability (KSE-G)
##############################
I8_D$SDPQ <- I8_D$SDPQ1+I8_D$SDPQ2+I8_D$SDPQ3
I8_D$SDNQ <- I8_D$SDNQ1+I8_D$SDNQ2+I8_D$SDNQ3

cor.test(I8_D$URGE, I8_D$SDPQ, use = "pairwise.complete.obs")
DE_URGE_cor <- c(DE_URGE_cor, cor.test(I8_D$URGE, I8_D$SDPQ, use =
"pairwise.complete.obs")[["estimate"]][["cor"]])
cor.test(I8_D$PREM, I8_D$SDPQ, use = "pairwise.complete.obs")
DE_PREM_cor <- c(DE_PREM_cor, cor.test(I8_D$PREM, I8_D$SDPQ, use =
"pairwise.complete.obs")[["estimate"]][["cor"]])
cor.test(I8_D$PERS, I8_D$SDPQ, use = "pairwise.complete.obs")
DE_PERS_cor <- c(DE_PERS_cor, cor.test(I8_D$PERS, I8_D$SDPQ, use =
"pairwise.complete.obs")[["estimate"]][["cor"]])
cor.test(I8_D$SENS, I8_D$SDPQ, use = "pairwise.complete.obs")
DE_SENS_cor <- c(DE_SENS_cor, cor.test(I8_D$SENS, I8_D$SDPQ, use =
"pairwise.complete.obs")[["estimate"]][["cor"]])

cor.test(I8_D$URGE, I8_D$SDNQ, use = "pairwise.complete.obs")
DE_URGE_cor <- c(DE_URGE_cor, cor.test(I8_D$URGE, I8_D$SDNQ, use =
"pairwise.complete.obs")[["estimate"]][["cor"]])
cor.test(I8_D$PREM, I8_D$SDNQ, use = "pairwise.complete.obs")
```

```
DE_PREM_cor <- c(DE_PREM_cor, cor.test(I8_D$PREM, I8_D$SDNQ, use =
"pairwise.complete.obs")[["estimate"]][["cor"]])
cor.test(I8_D$PERS, I8_D$SDNQ, use = "pairwise.complete.obs")
DE_PERS_cor <- c(DE_PERS_cor, cor.test(I8_D$PERS, I8_D$SDNQ, use =
"pairwise.complete.obs")[["estimate"]][["cor"]])
cor.test(I8_D$SENS, I8_D$SDNQ, use = "pairwise.complete.obs")
DE_SENS_cor <- c(DE_SENS_cor, cor.test(I8_D$SENS, I8_D$SDNQ, use =
"pairwise.complete.obs")[["estimate"]][["cor"]])

######################
#Employment status
######################
#1) employed
#2) self-employed
#3) out of work and looking for work
#4) out of work but not currently looking for work
#5) doing housework
#6) pupil/student
#7) apprentice/internship
#8) retired
#[9) none of what is mentioned above]
describe(I8_D$EMPL)
#unemployed vs. employed
I8_D$EMPL.empl <- recode(I8_D$EMPL, "3:4 = 1; 1:2 = 2; else = NA")
describe(I8_D$EMPL.empl)

cor.test(I8_D$URGE, I8_D$EMPL.empl, use = "pairwise.complete.obs")
DE_URGE_cor <- c(DE_URGE_cor, cor.test(I8_D$URGE, I8_D$EMPL.empl, use =
"pairwise.complete.obs")[["estimate"]][["cor"]])
cor.test(I8_D$PREM, I8_D$EMPL.empl, use = "pairwise.complete.obs")
DE_PREM_cor <- c(DE_PREM_cor, cor.test(I8_D$PREM, I8_D$EMPL.empl, use =
"pairwise.complete.obs")[["estimate"]][["cor"]])
cor.test(I8_D$PERS, I8_D$EMPL.empl, use = "pairwise.complete.obs")
DE_PERS_cor <- c(DE_PERS_cor, cor.test(I8_D$PERS, I8_D$EMPL.empl, use =
"pairwise.complete.obs")[["estimate"]][["cor"]])
cor.test(I8_D$SENS, I8_D$EMPL.empl, use = "pairwise.complete.obs")
DE_SENS_cor <- c(DE_SENS_cor, cor.test(I8_D$SENS, I8_D$EMPL.empl, use =
"pairwise.complete.obs")[["estimate"]][["cor"]])

########
#Income
########
cor.test(I8_D$URGE, I8_D$INCO, use = "pairwise.complete.obs")
```

```
DE_URGE_cor <- c(DE_URGE_cor, cor.test(I8_D$URGE, I8_D$INCO, use =
"pairwise.complete.obs")[["estimate"]][["cor"]])
cor.test(I8_D$PREM, I8_D$INCO, use = "pairwise.complete.obs")
DE_PREM_cor <- c(DE_PREM_cor, cor.test(I8_D$PREM, I8_D$INCO, use =
"pairwise.complete.obs")[["estimate"]][["cor"]])
cor.test(I8_D$PERS, I8_D$INCO, use = "pairwise.complete.obs")
DE_PERS_cor <- c(DE_PERS_cor, cor.test(I8_D$PERS, I8_D$INCO, use =
"pairwise.complete.obs")[["estimate"]][["cor"]])
cor.test(I8_D$SENS, I8_D$INCO, use = "pairwise.complete.obs")
DE_SENS_cor <- c(DE_SENS_cor, cor.test(I8_D$SENS, I8_D$INCO, use =
"pairwise.complete.obs")[["estimate"]][["cor"]])

########################
#Educational attainment
########################
cor.test(I8_D$URGE, I8_D$SCHO, use = "pairwise.complete.obs")
DE_URGE_cor <- c(DE_URGE_cor, cor.test(I8_D$URGE, I8_D$SCHO, use =
"pairwise.complete.obs")[["estimate"]][["cor"]])
cor.test(I8_D$PREM, I8_D$SCHO, use = "pairwise.complete.obs")
DE_PREM_cor <- c(DE_PREM_cor, cor.test(I8_D$PREM, I8_D$SCHO, use =
"pairwise.complete.obs")[["estimate"]][["cor"]])
cor.test(I8_D$PERS, I8_D$SCHO, use = "pairwise.complete.obs")
DE_PERS_cor <- c(DE_PERS_cor, cor.test(I8_D$PERS, I8_D$SCHO, use =
"pairwise.complete.obs")[["estimate"]][["cor"]])
cor.test(I8_D$SENS, I8_D$SCHO, use = "pairwise.complete.obs")
DE_SENS_cor <- c(DE_SENS_cor, cor.test(I8_D$SENS, I8_D$SCHO, use =
"pairwise.complete.obs")[["estimate"]][["cor"]])

#####
#Age
#####
cor.test(I8_D$URGE, I8_D$AGE, use = "pairwise.complete.obs")
DE_URGE_cor <- c(DE_URGE_cor, cor.test(I8_D$URGE, I8_D$AGE, use =
"pairwise.complete.obs")[["estimate"]][["cor"]])
cor.test(I8_D$PREM, I8_D$AGE, use = "pairwise.complete.obs")
DE_PREM_cor <- c(DE_PREM_cor, cor.test(I8_D$PREM, I8_D$AGE, use =
"pairwise.complete.obs")[["estimate"]][["cor"]])
cor.test(I8_D$PERS, I8_D$AGE, use = "pairwise.complete.obs")
DE_PERS_cor <- c(DE_PERS_cor, cor.test(I8_D$PERS, I8_D$AGE, use =
"pairwise.complete.obs")[["estimate"]][["cor"]])
cor.test(I8_D$SENS, I8_D$AGE, use = "pairwise.complete.obs")
DE_SENS_cor <- c(DE_SENS_cor, cor.test(I8_D$SENS, I8_D$AGE, use =
"pairwise.complete.obs")[["estimate"]][["cor"]])
```

```
#####
#Sex
#####
cor.test(I8_D$URGE, I8_D$SEX, use = "pairwise.complete.obs")
DE_URGE_cor <- c(DE_URGE_cor, cor.test(I8_D$URGE, I8_D$SEX, use =
"pairwise.complete.obs")[["estimate"]][["cor"]])
cor.test(I8_D$PREM, I8_D$SEX, use = "pairwise.complete.obs")
DE_PREM_cor <- c(DE_PREM_cor, cor.test(I8_D$PREM, I8_D$SEX, use =
"pairwise.complete.obs")[["estimate"]][["cor"]])
cor.test(I8_D$PERS, I8_D$SEX, use = "pairwise.complete.obs")
DE_PERS_cor <- c(DE_PERS_cor, cor.test(I8_D$PERS, I8_D$SEX, use =
"pairwise.complete.obs")[["estimate"]][["cor"]])
cor.test(I8_D$SENS, I8_D$SEX, use = "pairwise.complete.obs")
DE_SENS_cor <- c(DE_SENS_cor, cor.test(I8_D$SENS, I8_D$SEX, use =
"pairwise.complete.obs")[["estimate"]][["cor"]])

##############################################################################

##Similarity of correlations

#Urgency
URGE.cor <- data.frame(matrix(data=c(UK_URGE_cor,DE_URGE_cor),
nrow=length(dimension.matrix[[1]]), ncol=2, dimnames = dimension.matrix))
cor.test(URGE.cor$UK, URGE.cor$DE, use = "pairwise.complete.obs")

#Premeditation
PREM.cor <- data.frame(matrix(data=c(UK_PREM_cor,DE_PREM_cor),
nrow=length(dimension.matrix[[1]]), ncol=2, dimnames = dimension.matrix))
cor.test(PREM.cor$UK, PREM.cor$DE, use = "pairwise.complete.obs")

#Perseverance
PERS.cor <- data.frame(matrix(data=c(UK_PERS_cor,DE_PERS_cor),
nrow=length(dimension.matrix[[1]]), ncol=2, dimnames = dimension.matrix))
cor.test(PERS.cor$UK, PERS.cor$DE, use = "pairwise.complete.obs")

#Sensation Seeking
SENS.cor <- data.frame(matrix(data=c(UK_SENS_cor,DE_SENS_cor),
nrow=length(dimension.matrix[[1]]), ncol=2, dimnames = dimension.matrix))
cor.test(SENS.cor$UK, SENS.cor$DE, use = "pairwise.complete.obs")

##################################
#Step 5: Measurement Invariance
```

```
##################################

#Configural invariance
I8_MM_tau1 <- 'LV_URGE =~ c(a1, b1)*URGE1 + c(a1, b1)*URGE2
        LV_PREM =~ c(a2, b2)*PREM1 + c(a2, b2)*PREM2
        LV_PERS =~ c(a3, b3)*PERS1 + c(a3, b3)*PERS2
        LV_SENS =~ c(a4, b4)*SENS1 + c(a4, b4)*SENS2

        URGE1+PREM1+PERS1+SENS1 ~ c(0, 0)*1
        LV_URGE+LV_PREM+LV_PERS+LV_SENS ~ c(NA, NA)*1'

I8.fit1 <- sem(I8_MM_tau1, data = I8, group = "COUN", estimator = "mlr", missing = "fiml",
std.lv = FALSE)
summary(I8.fit1, standardized = T, fit.measures = T)

#Metric invariance (equals configural)
I8_MM_tau2 <- 'LV_URGE =~ c(a1, a1)*URGE1 + c(a1, a1)*URGE2
        LV_PREM =~ c(a2, a2)*PREM1 + c(a2, a2)*PREM2
        LV_PERS =~ c(a3, a3)*PERS1 + c(a3, a3)*PERS2
        LV_SENS =~ c(a4, a4)*SENS1 + c(a4, a4)*SENS2

        URGE1+PREM1+PERS1+SENS1 ~ c(0, 0)*1
        LV_URGE+LV_PREM+LV_PERS+LV_SENS ~ c(NA, NA)*1'

I8.fit2 <- cfa(I8_MM_tau2, data = I8, group = "COUN", estimator = "mlr", missing = "fiml",
std.lv=FALSE)
summary(I8.fit2, standardized = T, fit.measures = T)
anova(I8.fit2, I8.fit1)

#Scalar invariance
I8_MM_tau3 <- 'LV_URGE =~ c(a1, a1)*URGE1 + c(a1, a1)*URGE2
        LV_PREM =~ c(a2, a2)*PREM1 + c(a2, a2)*PREM2
        LV_PERS =~ c(a3, a3)*PERS1 + c(a3, a3)*PERS2
        LV_SENS =~ c(a4, a4)*SENS1 + c(a4, a4)*SENS2

        URGE1+PREM1+PERS1+SENS1 ~ c(0, 0)*1
        URGE2 ~ c(c1, c1)*1
        PREM2 ~ c(c2, c2)*1
        PERS2 ~ c(c3, c3)*1
        SENS2 ~ c(c4, c4)*1
        LV_URGE+LV_PREM+LV_PERS+LV_SENS ~ c(NA, NA)*1'
```

```
I8.fit3 <- cfa(I8_MM_tau3, data = I8, group = "COUN", estimator = "mlr", missing = "fiml",
std.lv=FALSE)
summary(I8.fit3, standardized = T, fit.measures = T)
anova(I8.fit3, I8.fit2)
I8.fit3.mi <- modindices(I8.fit3)
options(max.print=100000)
I8.fit3.mi #--> largest MI for URGE1 = 24.402

#Partial scalar invariance
I8_MM_tau3a <- 'LV_URGE =~ c(a1, a1)*URGE1 + c(a1, a1)*URGE2
        LV_PREM =~ c(a2, a2)*PREM1 + c(a2, a2)*PREM2
        LV_PERS =~ c(a3, a3)*PERS1 + c(a3, a3)*PERS2
        LV_SENS =~ c(a4, a4)*SENS1 + c(a4, a4)*SENS2

        PREM1+PERS1+SENS1 ~ c(0, 0)*1
        URGE2 ~ c(c1, c1)*1
        PREM2 ~ c(c2, c2)*1
        PERS2 ~ c(c3, c3)*1
        SENS2 ~ c(c4, c4)*1
        URGE1 ~ c(0, NA)*1
        LV_URGE+LV_PREM+LV_PERS+LV_SENS ~ c(NA, NA)*1'

I8.fit3a <- cfa(I8_MM_tau3a, data = I8, group = "COUN", estimator = "mlr", missing =
"fiml", std.lv=FALSE)
summary(I8.fit3a, standardized = T, fit.measures = T)
anova(I8.fit3a, I8.fit2)

#--> partial scalar invariance accepted

#Invariance of residual variances
I8_MM_tau4 <- 'LV_URGE =~ c(a1, a1)*URGE1 + c(a1, a1)*URGE2
        LV_PREM =~ c(a2, a2)*PREM1 + c(a2, a2)*PREM2
        LV_PERS =~ c(a3, a3)*PERS1 + c(a3, a3)*PERS2
        LV_SENS =~ c(a4, a4)*SENS1 + c(a4, a4)*SENS2

        PREM1+PERS1+SENS1 ~ c(0, 0)*1
        URGE2 ~ c(c1, c1)*1
        PREM2 ~ c(c2, c2)*1
        PERS2 ~ c(c3, c3)*1
        SENS2 ~ c(c4, c4)*1
        URGE1 ~ c(0, NA)*1
        LV_URGE+LV_PREM+LV_PERS+LV_SENS ~ c(NA, NA)*1
```

```
    URGE1 ~~ c(e1, e1)*URGE1
    URGE2 ~~ c(e2, e2)*URGE2
    PREM1 ~~ c(e3, e3)*PREM1
    PREM2 ~~ c(e4, e4)*PREM2
    PERS1 ~~ c(e5, e5)*PERS1
    PERS2 ~~ c(e6, e6)*PERS2
    SENS1 ~~ c(e7, e7)*SENS1
    SENS2 ~~ c(e8, e8)*SENS2
    '

I8.fit4 <- cfa(I8_MM_tau4, data = I8, group = "COUN", estimator = "mlr", missing = "fiml",
std.lv=FALSE)
summary(I8.fit4, standardized = T, fit.measures = T)
anova(I8.fit4, I8.fit3a)

#--> uniqueness invariance accepted

#Latent variance invariance
I8_MM_tau5 <- 'LV_URGE =~ c(a1, a1)*URGE1 + c(a1, a1)*URGE2
    LV_PREM =~ c(a2, a2)*PREM1 + c(a2, a2)*PREM2
    LV_PERS =~ c(a3, a3)*PERS1 + c(a3, a3)*PERS2
    LV_SENS =~ c(a4, a4)*SENS1 + c(a4, a4)*SENS2

    LV_URGE ~~ c(b1, b1)*LV_URGE
    LV_PREM ~~ c(b2, b2)*LV_PREM
    LV_PERS ~~ c(b3, b3)*LV_PERS
    LV_SENS ~~ c(b4, b4)*LV_SENS

    PREM1+PERS1+SENS1 ~ c(0, 0)*1
    URGE2 ~ c(c1, c1)*1
    PREM2 ~ c(c2, c2)*1
    PERS2 ~ c(c3, c3)*1
    SENS2 ~ c(c4, c4)*1
    URGE1 ~ c(0, NA)*1
    LV_URGE+LV_PREM+LV_PERS+LV_SENS ~ c(NA, NA)*1

    URGE1 ~~ c(e1, e1)*URGE1
    URGE2 ~~ c(e2, e2)*URGE2
    PREM1 ~~ c(e3, e3)*PREM1
    PREM2 ~~ c(e4, e4)*PREM2
    PERS1 ~~ c(e5, e5)*PERS1
    PERS2 ~~ c(e6, e6)*PERS2
    SENS1 ~~ c(e7, e7)*SENS1
```

```
	SENS2 ~~ c(e8, e8)*SENS2
	'

I8.fit5 <- cfa(I8_MM_tau5, data = I8, group = "COUN", estimator = "mlr", missing = "fiml", std.lv=FALSE)
summary(I8.fit5, standardized = T, fit.measures = T)
anova(I8.fit5, I8.fit4)
#I8.fit5.mi <- modindices(I8.fit5)
#options(max.print=100000)
#I8.fit5.mi

#--> invariance of latent variances is rejected (see also latent variance values from uniqueness model)

#Latent covariance invariance
I8_MM_tau6 <- 'LV_URGE =~ c(a1, a1)*URGE1 + c(a1, a1)*URGE2
	LV_PREM =~ c(a2, a2)*PREM1 + c(a2, a2)*PREM2
	LV_PERS =~ c(a3, a3)*PERS1 + c(a3, a3)*PERS2
	LV_SENS =~ c(a4, a4)*SENS1 + c(a4, a4)*SENS2

	PREM1+PERS1+SENS1 ~ c(0, 0)*1
	URGE2 ~ c(c1, c1)*1
	PREM2 ~ c(c2, c2)*1
	PERS2 ~ c(c3, c3)*1
	SENS2 ~ c(c4, c4)*1
	URGE1 ~ c(0, NA)*1
	LV_URGE+LV_PREM+LV_PERS+LV_SENS ~ c(NA, NA)*1

	URGE1 ~~ c(e1, e1)*URGE1
	URGE2 ~~ c(e2, e2)*URGE2
	PREM1 ~~ c(e3, e3)*PREM1
	PREM2 ~~ c(e4, e4)*PREM2
	PERS1 ~~ c(e5, e5)*PERS1
	PERS2 ~~ c(e6, e6)*PERS2
	SENS1 ~~ c(e7, e7)*SENS1
	SENS2 ~~ c(e8, e8)*SENS2

	LV_URGE ~~ c(f1, f1)*LV_PREM
	LV_URGE ~~ c(f2, f2)*LV_PERS
	LV_URGE ~~ c(f3, f3)*LV_SENS
	LV_PREM ~~ c(f4, f4)*LV_PERS
	LV_PREM ~~ c(f5, f5)*LV_SENS
	LV_PERS ~~ c(f6, f6)*LV_SENS
```

```
	'

I8.fit6 <- cfa(I8_MM_tau6, data = I8, group = "COUN", estimator = "mlr", missing = "fiml", std.lv=FALSE)
summary(I8.fit6, standardized = T, fit.measures = T)
anova(I8.fit6, I8.fit4)
#I8.fit6.mi <- modindices(I8.fit6)
#options(max.print=100000)
#I8.fit6.mi

#--> invariance of latent covariances is rejected (see also latent covariance values from uniqueness model)


#Latent mean invariance
I8_MM_tau7 <- 'LV_URGE =~ c(a1, a1)*URGE1 + c(a1, a1)*URGE2
	LV_PREM =~ c(a2, a2)*PREM1 + c(a2, a2)*PREM2
	LV_PERS =~ c(a3, a3)*PERS1 + c(a3, a3)*PERS2
	LV_SENS =~ c(a4, a4)*SENS1 + c(a4, a4)*SENS2

	PREM1+PERS1+SENS1 ~ c(0, 0)*1
	URGE2 ~ c(c1, c1)*1
	PREM2 ~ c(c2, c2)*1
	PERS2 ~ c(c3, c3)*1
	SENS2 ~ c(c4, c4)*1
	URGE1 ~ c(0, NA)*1

	LV_URGE ~ c(d1, d1)*1
	LV_PREM ~ c(d2, d2)*1
	LV_PERS ~ c(d3, d3)*1
	LV_SENS ~ c(d4, d4)*1

	URGE1 ~~ c(e1, e1)*URGE1
	URGE2 ~~ c(e2, e2)*URGE2
	PREM1 ~~ c(e3, e3)*PREM1
	PREM2 ~~ c(e4, e4)*PREM2
	PERS1 ~~ c(e5, e5)*PERS1
	PERS2 ~~ c(e6, e6)*PERS2
	SENS1 ~~ c(e7, e7)*SENS1
	SENS2 ~~ c(e8, e8)*SENS2
	'
```

```
I8.fit7 <- cfa(I8_MM_tau7, data = I8, group = "COUN", estimator = "mlr", missing = "fiml",
std.lv=FALSE)
summary(I8.fit7, standardized = T, fit.measures = T)
anova(I8.fit7, I8.fit4)
#I8.fit7.mi <- modindices(I8.fit7)
#options(max.print=100000)
#I8.fit7.mi

#--> invariance of latent means is rejected (see also latent mean values from uniqueness
model)
```
